# “What Else Could It Be?” A Scoping Review of Questions for Patients to Ask Throughout the Diagnostic Process

**DOI:** 10.1097/PTS.0000000000001273

**Published:** 2024-09-11

**Authors:** Mary A. Hill, Tess Coppinger, Kimia Sedig, William J. Gallagher, Kelley M. Baker, Helen Haskell, Kristen E. Miller, Kelly M. Smith

**Affiliations:** * University of Toronto, Institute of Health Policy, Management & Evaluation, Toronto, Canada; † Michael Garron Hospital, Toronto East Health Network, Toronto, Canada; ‡ Georgetown University School of Medicine, Washington, District of Columbia; § National Center for Human Factors in Healthcare, MedStar Health, Washington, District of Columbia; ‖ Mothers Against Medical Error, Columbia, South Carolina.

**Keywords:** diagnostic safety, question prompt list, communication, patient engagement

## Abstract

**Background:**

Diagnostic errors are a global patient safety challenge. Over 75% of diagnostic errors in ambulatory care result from breakdowns in patient-clinician communication. Encouraging patients to speak up and ask questions has been recommended as one strategy to mitigate these failures.

**Objectives:**

The goal of the scoping review was to identify, summarize, and thematically map questions patients are recommended to ask during ambulatory encounters along the diagnostic process. This is the first step in a larger study to co-design a patient-facing question prompt list for patients to use throughout the diagnostic process.

**Methods:**

Medline and Google Scholar were searched to identify question lists in the peer-reviewed literature. Organizational websites and a search engine were searched to identify question lists in the gray literature. Articles and resources were screened for eligibility and data were abstracted. Interrater reliability (K = 0.875) was achieved.

**Results:**

A total of 5509 questions from 235 resources met inclusion criteria. Most questions (*n* = 4243, 77.02%) were found in the gray literature. Question lists included an average of 23.44 questions. Questions were most commonly coded along the diagnostic process categories of treatment (2434 questions from 250 resources), communication of the diagnosis (1160 questions, 204 resources), and outcomes (766 questions, 172 resources).

**Conclusions:**

Despite recommendations for patients to ask questions, most question prompt lists focus on later stages of the diagnostic process such as communication of the diagnosis, treatment, and outcomes. Further research is needed to identify and prioritize diagnostic-related questions from the patient perspective and to develop simple, usable guidance on question-asking to improve patient safety across the diagnostic continuum.

Diagnostic error, defined as the “failure to a) establish an accurate and timely explanation of the patient’s health problem or b) communicate that explanation to the patient,” is a global patient safety challenge.^1–3^ Evidence suggests diagnostic error accounts for more serious harm than any other type of medical error^4^ and leads to approximately 795,000 serious harm incidents in the United States each year.^5^ In the ambulatory care setting, 1 in 20 patients experiences a diagnostic error.^6^

Communication breakdowns between patients and clinicians have consistently been shown to be a point of failure in the diagnostic process^2,7–10^ and are a significant threat to patient safety in ambulatory care.^11^ Breakdowns in clinician-patient communication, such as misunderstandings and failures to effectively relay critical information,^12–14^ have been identified as common and avoidable sources of error.^2,10^ Strategies to enhance patient-clinician communication have been recommended to support patient engagement and improve diagnostic safety. Such strategies include patient access to clinical notes,^15^ use of agenda-setting tools,^16–19^ and promotion of question asking through patient-facing question prompt lists (QPLs).^20–23^

The National Academies of Science, Engineering, and Medicine (NASEM), in their landmark report on improving diagnosis, recognized the importance of patients asking questions to improve diagnostic safety. NASEM recommends that patients should “*speak up and ask questions*” about their care, and providers should “*create a safe space and opportunity for patients to speak up during their appointments.*”^2^ Asking questions allows patients to be more engaged in shared decision making and fill in gaps in their understanding related to their diagnosis, prognosis, and plans of care.^24–26^ Question asking has been shown to increase through the use of QPLs, particularly when they are provided at the point of contact and endorsed by the clinician.^20–23,27^ Most patients and caregivers search for health information online,^28–31^ regardless of health condition, sex, and race,^28,29,31^ and there are many QPLs and other question-asking resources available online for patients to use.^32^

Prior review studies have examined the availability and readability of QPLs,^32^ as well as their use and effectiveness.^20^ However, to our knowledge, no study has reviewed the available patient-facing QPLs specifically through the lens of the NASEM diagnostic process. In this scoping review, our objective was to identify and summarize questions for patients and their families to ask during ambulatory clinical encounters and to map the questions to the diagnostic process. Specifically, we aimed to address the following research questions: (1) what patient-facing QPLs are available to facilitate patient-provider communication during ambulatory care encounters and (2) where do the questions in the QPLs fall along the diagnostic process? We were also interested in characteristics of available QPLs, such as the target audience(s), condition(s), and setting(s), and the number of questions included. Our results will inform the design of a patient-friendly, effective, and accessible patient-facing QPL, with the goal of improving diagnostic safety.

## METHODS

### Design

We conducted a scoping review to identify literature on patient question-asking for improved patient-clinician communication during the diagnostic process. Our review was conducted using Arskey and O’Malley’s scoping review framework^33^ and in accordance with the Preferred Reporting Items for Systematic Reviews and Meta-analyses Extension for Scoping Reviews.^34,35^ We selected a scoping review because it supports the inclusion of both peer-reviewed and gray literature, and prior studies on patient engagement strategies suggest that much of the work in this field remains in the gray literature.^11^ The literatures were reviewed and abstracted between July 13, 2022, and February 3, 2023.

### Search Strategy

We searched electronic databases for peer-reviewed literature and systematically scanned relevant websites for gray literature. The search was designed to capture QPLs, including those embedded in agenda-setting tools, patient-education materials, and other resources, that provide recommendations for questions to ask during clinical encounters. We also hand searched the table of contents of domain-specific, nonindexed sources and reference lists of peer-reviewed literature.

To identify resources from the published literature, we searched Medline and Google Scholar using broad search terms relating to question-asking, agenda-setting, and question prompt lists. We identified search terms by reviewing 4 peer-reviewed manuscripts related to question-asking, agenda-setting, and prompt lists and by capturing MeSH terms under which these articles were indexed.^15,19,24,25^ We used text word terms and phrases where no MeSH terms met the intent of the search. The nonindexed portion of Medline was searched separately with a text-only variant of the search. A full list of search terms is available in Appendix A, http://links.lww.com/JPS/A644.

Internet sources most commonly used by patients seeking health information include search engines (e.g., Google), medical societies/organizations, health systems, news organizations, and other health-related websites, such as WebMD and other patient-led websites.^14,15,18^ With this in mind, we developed a gray literature search strategy for the following sources (full list available in Appendix A, http://links.lww.com/JPS/A644):

- Search engine
- Relevant medical organizations, associations, and societies
- Health systems
- Patient and diagnostic safety organizations
- Relevant government agency websites
- Online news and magazine sources
- Other health-related websites (e.g., WedMD, Verywell Health)

The search engine and targeted websites were searched systematically.^36^ DuckDuckGo (DDG) was chosen as the search engine to reduce bias in the results returned. Search phrases such as “*questions to ask my doctor*,” “*patient questions*,” or “*question prompt lists*” were entered into DDG and the first 10 pages (100 results) were reviewed for relevant material. We established a “cutoff ” point of 100 results per search term/phrase, as recommended in the literature, because each search returned thousands of results and it would have been impossible to review them all.^36^ Targeted website searches began with hand-searching website home pages for potentially relevant resources. Toolbars were then reviewed for research, publication, or tool links where patient questions may have been housed. Finally, the website’s search engines were searched using the same strategy used for DDG. Website searchers were trained on standards for searching, operational definitions, and goals of the research questions.

### Selection Criteria

**FIGURE 1.**
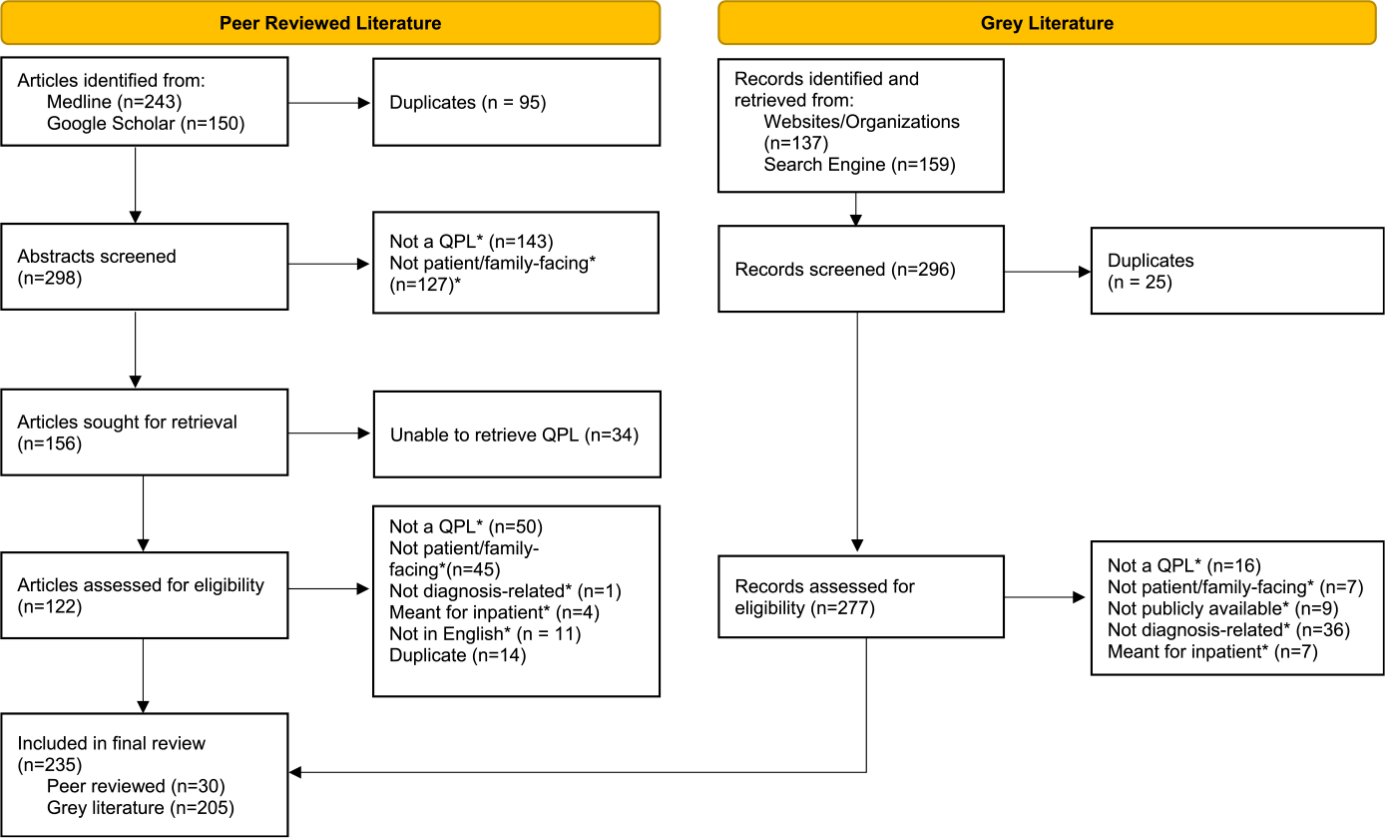
The Preferred Reporting Items for Systematic Reviews and Meta-analyses flow diagram for the scoping review of the literature “What else could it be?” A scoping review of questions for patients to ask throughout the NASEM diagnostic process.^35^

Articles and resources were included in this study if they were available in English, included a QPL with at least one diagnosis-related question, were patient-facing, and focused on ambulatory encounters. Articles and resources were excluded if the full QPL was not available; articles or resources that only included partial QPLs or sections of the QPL were excluded. Included publications (articles, abstracts, and theses/dissertations) were published between January 1, 2015, and February 3, 2023. This date range was informed by the study team’s knowledge that research related to engaging patients in diagnostic communication has escalated since the NASEM report was published in 2015.^2^ Figure 1 provides an overview of the flow of articles selected for final inclusion in the study.

Three members of the project team pilot tested the inclusion/exclusion criteria with a sample of abstracts retrieved from the peer and gray literature searches. Interrater reliability (K = 0.875) was achieved. Research assistants worked with oversight from the senior author to complete screening and met weekly to review the findings.

### Data Abstraction

Following the deduplication and initial screen, our team retrieved full-text articles of the peer-reviewed literature resources. Full-text articles that were not available to the study team were excluded from further analysis. We developed a study-specific codebook for multistage template analysis using inductive and deductive strategies.^37^ The codebook templates were designed to extract general information from the articles/resources including bibliographic information; target audience(s), condition(s) and setting (s); and the number of questions in each resource (see Appendix B, http://links.lww.com/JPS/A645).

Templates also allowed for mapping of individual questions to the relevant step(s) in the NASEM diagnostic process: (1) pre-engagement, (2) patient engagement with the health system, (3) clinical history/interview, (4) physical exam, (5) diagnostic testing, (6) referrals and consultations, (7) communication of the diagnosis, (8) treatment, and (9) outcomes.^2^ The NASEM diagnostic framework does not include definitions for each step. To address this, the authors developed definitions with input from the larger research team. The steps in the diagnostic process were not mutually exclusive, and many questions were coded as more than 1 step. The codebook is available in Appendix B, http://links.lww.com/JPS/A645.

Questions unrelated to the diagnostic process, questions for patients to ask themselves (self-reflective), or example questions that doctors may ask their patients were excluded from the question mapping exercise. Questions related to pregnancy and or/fertility were also excluded as these topics were outside the scope of the grant under which this study was performed.

All abstractors were trained on the project goals and oriented to the codebook. If an abstractor was unsure of how to code a specific question, they marked the question for adjudication. Team code reviews and adjudication by the senior author were completed weekly. Qualitative Statistical Software NVivo version 13 (Lumivero, Denver, CO) was used to complete template coding and analysis.

### Collating, Summarizing, and Reporting the Results

We used frequencies and percentages to summarize quantitative data collected on bibliographic information and template-coded items. Means and medians were also used to summarize quantitative data where appropriate. As steps in the diagnostic process were not mutually exclusive, we did not report percentages or conduct analysis beyond basic frequencies for these items.

Arksey and O’Malley recommend a final phase in their framework for scoping reviews: to consult with stakeholders on the resources identified and the results of the review.^33^ With this in mind, the research team frequently consulted with our laboratory’s investigator team monthly to gain feedback on the scope of work, search strategy, and how we chose to operationalize steps in the diagnostic process for coding.

## RESULTS

The literature searches returned 393 peer-reviewed articles and 296 gray literature resources. After removing duplicates and conducting screening, 122 peer-reviewed articles and 277 gray literature resources were assessed for eligibility. Approximately 41% (n = 50) of peer-reviewed articles assessed did not include a full QPL and nearly 37% (n = 45) included QPLs that were not patient facing; 13% (n = 36) of gray literature resources were excluded as the QPLs were not related to diagnosis. The final database included 235 articles and resources (peer-reviewed = 30; gray = 205). See Appendix C, http://links.lww.com/JPS/A646, for full list of included articles and resources.

### Number of Questions

A total of 5509 individual questions were identified, including 1266 (22.98%) questions from the peer-reviewed literature and 4243 (77.02%) questions from the gray literature. Question prompt lists included 3–113 questions each (mean = 23.4; median = 16). Question prompt lists from the peer-reviewed literature ranged from 4 to 112 questions (mean = 42.4; median = 29.5) and gray literature QPLs ranged from 3 to 113 questions (mean = 20.7; median = 15).

### Resource-Level Characteristics

In all, 199 QPLs were meant for use when seeking a diagnosis for new or acute symptoms, and 75 were meant for use when diagnosing changes in existing or ongoing diagnoses. Most QPLs targeted a specific diagnosis or condition (n = 170, 72.3%) such as cancer, heart disease, or behavioral health conditions. A total of 27 resources (11.5%) focused on specific age groups including pediatrics, adults, and older adults. Another 13 resources focused on “men” (n = 3) or “women,” (n = 10) although most did not explicitly state whether they were meant for use by cisgender and/or transgender individuals. Because of the nature of the questions (e.g., questions about menopause, ovarian cancer, prostate cancer), it was interpreted that these QPLs were intended for individuals of cisgender male and female sex. A total of 23 QPLs included questions specifically for caregivers or family members and 5 (8.9%) were designed for Black or African American individuals. There was 1 QPL each for individuals with religious/spiritual needs, economically and racially/ethnically diverse individuals, individuals with below average health literacy, and individuals with Medicare. A full list of question prompt lists and their characteristics can be found in Appendix D, http://links.lww.com/JPS/A647

### Questions Mapped to the Diagnostic Process

Within the diagnostic process, treatment was the most common category for questions, with 2212 questions from 219 QPLs. Questions to engage patients in shared decision making around treatment such as, “*What are the benefits versus risks of each treatment option?*” *(G014),* were common throughout the literature. After treatment, the next most common categories were communication of the diagnosis (1090 questions, 190 QPLs) and outcomes (703 questions, 167 QPLs). The least common category was physical exam (50 questions, 16 QPLs). Table 1 provides a summary of the coding results with examples of questions that fell under each category.

## DISCUSSION

This study highlights the vast number of questions available for patients in the published and gray literature and extends our current understanding of question asking to the diagnostic process. Question prompt lists in this study tended to focus on the later steps within the diagnostic process during the treatment, communication of the diagnosis, and outcomes stages. This is not surprising as improving communication and shared decision making (SDM) between patients and providers QPLs are important to the patient’s understanding of the diagnosis and related uncertainty,^38–40^ developing treatment plans,^41–45^ and ensuring positive patient outcomes such as treatment adherence and disease management.^46–49^ Improving communication and SDM are often the goals of QPLs when they are created.^21,22,26,32^ However, the relative dearth of questions in the remainder of the diagnostic process may limit patient engagement during the earlier processes of information gathering, integration, and hypothesis generation.

Many of the questions captured in this review were double-coded as more than 1 category. Categories that were frequently double-coded included treatments and outcomes, outcomes and pre-engagement, and testing and treatments. This is likely because the diagnostic process is a complex, iterative process that is not linear.^2^ Patients and their care teams may go through several rounds of information gathering, hypothesis generation, and treatments throughout their diagnostic journey, which may require patients to ask the same questions more than once as more information is learned.^2^

Our results are consistent with other studies findings on the burdens of question asking. One study focused on QPLs available on the Internet and found 173 unique QPLs ranging from 1 to more than 200 questions. Patients have reported that they often do not know what to ask or how to ask it,^26^ and QPLs may be useful to help close patient-provider communication gaps impacting patient safety along the diagnostic process. However, providing patients with too many question options can be overwhelming.^26^ With so many existing resources, it could be difficult for a patient to sift through the available QPLs. Even individual resources included an overwhelming number of questions. We found the average number of questions per QPL to be 21, too many questions for a patient to ask or a care provider to answer in a standard 10- to 20-minute ambulatory appointment. One study found that even 11 questions were burdensome for a medically underserved population.^22^ More research is needed to develop simple, usable guidance to encourage patients to ask questions, leveraging the abundant available resources, throughout the diagnostic process.

### Limitations

There are 3 primary limitations to this study. First, there was an enormous amount of literature related to our research question, which was impossible to capture in its entirety. We established a “cutoff point” beyond which no new resources were included in our study. This could have prevented us from capturing relevant resources that patients are using to help inform their question-asking behaviors in clinical encounters. This limitation is mitigated by the prioritization scheme in our search strategy and by the volume of resources we were able to include. Despite this limitation, we assert that our study provides a thorough representation of the question-asking resources for patients that they would be likely to locate through a standard web search. Second, our study excluded the widely recognized Agency for Healthcare Research and Quality “Question Builder App” from its review as it met the exclusion criterion of having no publicly available full list of questions. Another limitation was the lack of detail provided in the NASEM report about each stage in the diagnostic process. Our research team addressed this by codesigning patient-centered definitions and examples in consultation with our broader team of experts to map questions to the diagnostic process.

## CONCLUSIONS

While patients are encouraged to speak up and ask questions to improve their diagnostic safety, there are a vast number of questions in the literature for patients to sift through to identify questions relevant to their diagnostic journeys. The literature is replete with questions for patients to ask about the later stages of the diagnostic process from communication of the diagnosis to monitoring of treatments and outcomes. Future research is needed to identify and prioritize the most important questions, from the patient perspective, to ask at each stage of the diagnostic process. Such research, combined with the results of this study, could be used to inform the design of patient-facing guidance on question asking to effectively enhance patient-provider communication around diagnosis.

## Supplementary Material

AppendixC

AppendixA

AppendixB

AppendixD

## Figures and Tables

**TABLE 1.** Results Table With Definitions of Diagnostic Process Categories and Examples

Diagnostic Process Steps	Definition	N Questions*	Examples

Pre-engagement	Questions for the patient to ask prior to accessing the health care system for a new diagnosis or condition. This may include questions about new appointments, identifying symptoms, or other questions to help patients make decisions about when, how, or where to access care.	295	*What should I do if I have symptoms of COVID-19? (G049)* *What are the warning signs of heart disease and stroke? (G063)* *If I am worried about how my baby is acting, who should I tell? (P039)*
Patient engagement in the health system	Questions for the patient to ask when accessing the health care system. This includes questions related to insurance, cost of care, what/who to bring with you to your appointment, and the care team’s contact information.	208	*Can a support person (such as a partner, parent or friend) be present? (G205)* *What should I do if I have trouble paying for my medical care? (G296)*
Clinical history & interview	Questions for the patient to ask during a discussion of their family history, social history, medical history, and other history of events leading to the health problem.	205	*Can I discuss my lack of appetite, difficulty eating and weight loss? (P373)* *Based on my family history and lifestyle, am I at risk for the following cancers? (G135)* *Are you aware of each of the medications that I am taking? (G014)*
Physical exam	Questions for the patient to ask related to the routine physical exam. This includes exam of blood pressure, weight, temperature, etc.	50	*Is my weight at a healthy level for my height and age? (G004)* *What should my blood pressure be? (P187)*
Diagnostic testing	Questions for the patient to ask about tests that go beyond the routine physical exam. These tests may include mammograms, colonoscopies, blood tests, stool tests, etc.	459	*What information will this test provide? (G232)* *Can you explain my pathology report (laboratory test results) to me? (P325)* *Do I need to undergo any additional tests? (G112)*
Referrals and consultations	Questions for patients to ask about referrals to providers outside of their typical care team. This may include condition-specific specialists (cardiologists, oncologists, etc.), diagnostic specialists (radiologists, pathologists, etc.), or other doctors consulting on the patient’s care (other primary care physicians, internists, dieticians, etc.)	207	*Where can I get a second opinion about this surgery? (G004)* *Should I see a dermatologist? (G089)* *Can you help me formulate questions that I may wish to ask other doctors and cancer specialists? (P007)*
Communication of the diagnosis	Questions for patients to ask about the provider’s explanation of the health problem (diagnosis) or working diagnosis. Includes questions about the patient’s prognosis, alternative diagnoses (differentials), explaining the diagnosis in the patient’s own terms, and where to get more information.	1090	*What kind of cancer do I have? (G086)* *Is there any written information about ADHD that you could provide me with? (P304)* *What else can it be? (G170)*
Treatment	Questions to help engage patients in shared decision making around treatment and treatment goals, or decision not to treat. This could include questions about therapies, medicines, surgeries, or other approaches to improve or manage a patient’s condition, including social prescribing.	2212	*Are there are guidelines for treating this kind of cancer? (P061)* *What will happen if I decide not to get treatment right now? (G027)* *Do comorbidities affect treatment or are they made worse by treatment? (P373)*
Outcomes	Questions about patient outcomes and postengagement work after the treatment plan has been decided on. Also includes questions to help patients make decisions about when, how, or where to re-engage in care.	703	*If my symptoms worsen, what should I do on my own? When should I contact you? (G014)* *How will I know if my child is making progress? (G046)* *What can I do before my next appointment? (G062)*

* Categories were not mutually exclusive.
