# Supplementary material for: “What Else Could It Be?” A Scoping Review of Questions for Patients to Ask Throughout the Diagnostic Process
Source: J Patient Saf. Author manuscript; Available in PMC 2025 Feb 7. (PMC11803640; doi:10.1097/PTS.0000000000001273)
Supplement: AppendixC [file NIHMS2048852-supplement-AppendixC.docx]

| **Appendix C. Articles and Resources for Final Inclusion** | | | | | |
| --- | --- | --- | --- | --- | --- |
| **Study ID** | **Author(s)** | **Year/ID** | **Journal or Organization** | **Article/Resource Title** | **DOI or URL** |
| P028 | Ahmed R, et al. | 2016;19(2): 234-52 | Health Expect | Development and validation of a question prompt list for parents of children with attention-deficit/hyperactivity disorder: a Delphi study | 10.1111/hex.12341 |
| G153 | Allen J, et al. | N/A | Premier Health | What 10 questions can help start a conversation between a patient and medical provider? | https://www.premierhealth.com/your-health/health-topics/health-literacy/health-literacy-faq/what-10-questions-can-help-start-a-conversation-between-a-patient-and-medical-provider- |
| G258 | Alli RA. | 2021 | WebMD® | Asthma in Children: 12 Questions to Ask Your Doctor | https://www.WebMD®.com/asthma/asthma-in-children-12-questions-to-ask-your-doctor |
| G234 | Anderson J. | 2020 | VeryWell Health | 8 Questions to Ask Your Healthcare Provider After a Celiac Disease Diagnosis | https://www.verywellhealth.com/celiac-disease-diagnosis-questions-to-ask-afterward-562713 |
| G088 | Anderson LA, et al. | 2022 | Health Central | 10 Questions to Ask Your Doctor about RA | https://www.healthcentral.com/article/10-questions-to-ask-your-doctor-about-ra |
| G039 | Anderson LA. | 2022 | Drugs.com | Multiple Sclerosis: Top Questions to Ask Your Doctor | https://www.drugs.com/slideshow/multiple-sclerosis-questions-1091 |
| G102 | Ashley WWJ. | 2011 | Chicago Health | Brain Aneurysms: Questions to Ask Your Doctor | https://chicagohealthonline.com/brain-aneurysms-questions-to-ask-your-doctor/ |
| G268 | Bandukwala NQ. | 2021 | WebMD® | Male Urinary Incontinence: 12 Questions to Ask Your Doctor | https://www.WebMD®.com/urinary-incontinence-oab/male-urinary-incontinence-12-questions-to-ask-your-doctor |
| G123 | Barden A. | 2022 | All About Vision | Questions to ask your child’s eye doctor | https://www.allaboutvision.com/eye-exam/child-eye-doctor-questions/ |
| P011 | Barton E, et al. | 2020;103(3):505-513 | Patient Educ Couns | The influence of a question prompt list on patient-oncologist information exchange in an African-American population | 10.1016/j.pec.2019.09.020 |
| G242 | Beckerman J. | 2020 | WebMD® | 10 Questions to Ask Your Doctor About Heart Failure | https://www.WebMD®.com/heart-disease/heart-failure/10-important-questions-ask-doctor-heart-failure |
| G255 | Beckerman J. | 2021 | WebMD® | 10 Questions to Ask Your Doctor About Stroke | https://www.WebMD®.com/stroke/guide/questions-doctor-stroke |
| G275 | Beckerman J. | 2022 | WebMD® | Atrial Fibrillation | https://www.WebMD®.com/a-to-z-guides/ddg-afib-treatment |
| G280 | Beckerman J. | 2021 | WebMD® | What questions should you ask your doctor about pulmonary arterial hypertension? | https://www.WebMD®.com/lung/qa/what-questions-should-you-ask-your-doctor-about-pulmonary-arterial-hypertension |
| P064 | Berger Z, et al. | 2019;3(53):1-6 | J Patient Rep Outcomes | Feasibility and perception of a question prompt list in outpatient cancer care | 10.1186/s41687-019-0145-y |
| G010 | Bertone H. | 2021 | Pink Fortitude | 25 Questions to Ask Your Doctor When You’ve Been Diagnosed with Autoimmune Disease | https://pinkfortitude.com/questions-to-ask-your-doctor/ |
| P003 | Best M, et al. | 2019;102(12): 2208-2213 | Patient Educ Couns | How and how much is spirituality discussed in palliative care consultations for advanced cancer patients with and without a question prompt list? | 10.1016/j.pec.2019.06.016 |
| G244 | Bhandari S. | 2021 | WebMD® | Questions to Ask Your Doctor About Schizophrenia | https://www.WebMD®.com/schizophrenia/10-questions-to-ask-your-doctor-about-schizophrenia |
| G267 | Bhandari S. | 2020 | WebMD® | 10 Questions to Ask Your Doctor About Treatment-Resistant Depression | https://www.WebMD®.com/depression/10-questions-ask-doctor-treatment-resistant-depression |
| P023 | Bottacini A, et al. | 2017;7(8):e015079 | BMJ Open | The involvement of early stage breast cancer patients during oncology consultations in Italy: a multi-centred, randomized controlled trial of a question prompt sheet versus question listing | 10.1136/bmjopen-2016-015079 |
| P007 | Bouleuc C, et al. | 2021;61(2):331-341.e8 | J Pain Symptom Manage | A question prompt list for advanced cancer patients promoting advance care planning: a french randomized trial | 10.1016/j.jpainsymman.2020.07.026 |
| G135 | Bramlet K. | 2016 | MD Anderson Cancer Center | Questions to ask your doctor about cancer prevention | https://www.mdanderson.org/publications/focused-on-health/questions-to-ask-doctor-cancer-prevention.h29Z1590624.html |
| G282 | Brennan D. | 2020 | WebMD® | Questions to Ask Your Child’s Oncologist | https://www.WebMD®.com/cancer/questions-childs-oncologist |
| G160 | Candclario C. | 2021 | PureWow | 8 Questions BIPOC Patients Should Always Ask Their Doctor, According to a Black Physician | https://www.purewow.com/wellness/questions-bipoc-patients-should-ask-their-doctor |
| G227 | Chen D. | 2019 | TED | Prevent unnecessary medical care — by asking your doctor these 4 questions first | https://ideas.ted.com/prevent-unnecessary-medical-care-by-asking-your-doctor-these-4-questions-first/ |
| G067 | Curtis L, et al. | 2022 | Verywell Health | Lung Cancer Questions to Ask Your Oncologist | https://www.verywellhealth.com/lung-cancer-questions-5219014 |
| G065 | Daniels J. | 2022 | BlackDoctor, Inc. | Diabetes: Questions to Ask Your Doctor | https://blackdoctor.org/diabetes-questions-to-ask-your-doctor/2/ |
| G270 | Davis JL, et al. | 2021 | WebMD® | 10 Questions to Ask Your Doctor About Hormone Therapy During Menopause | https://www.WebMD®.com/menopause/guide/10-questions-hormone-therapy-during-menopause |
| G273 | Davis JL, et al. | 2020 | WebMD® | 10 Questions to Ask Doctor About Bipolar Disorder | https://www.WebMD®.com/bipolar-disorder/10-questions-ask-about-treating-bipolar-mania |
| G257 | Davis JL. | 2021 | WebMD® | Questions to Ask Your Doctor About Depression Symptoms | https://www.WebMD®.com/depression/features/questions-to-ask-your-doctor-about-depression-symptoms |
| G246 | DerSarkissian C. | 2020 | WebMD® | Questions to Ask Your Doctor About Osteoarthritis | https://www.WebMD®.com/osteoarthritis/questions-for-doctor |
| G248 | DerSarkissian C. | 2022 | WebMD® | 10 Questions to Ask Your Doctor About Asthma | https://www.WebMD®.com/asthma/guide/questions-doctor-asthma |
| G281 | DerSarkissian C. | 2020 | WebMD® | Multiple Sclerosis: Now What? | https://www.WebMD®.com/multiple-sclerosis/questions-about-multiple-sclerosis |
| P187 | Dutton, K. | 2021 | ProQuest Dissertations Publishing | Discussion of Patient Questions During Clinic Visits to Improve Shared Decision-Making | N/A |
| P061 | Eggly S, et al. | 2017;100(5):818-826 | Patient Educ Couns | Randomized trial of a question prompt list to increase patient active participation during interactions with black patients and their oncologists | 10.1016/j.pec.2016.12.026 |
| G109 | Eidelson SG. | 2019 | Spine Universe | Spine Surgery: Questions to Ask Your Doctor | https://www.spineuniverse.com/treatments/surgery/spine-surgery-questions-ask-your-doctor |
| P025a; P025b | Ekberg S, et al. | 2020;34(3):291-299 | Palliat Med | Finding a way with words: Delphi study to develop a discussion prompt list for paediatric palliative care | 10.1177/0269216319888988 |
| G228 | Epstein HM. | 2019 | Society to Improve Diagnosis in Medicine | Ask these key questions when you get a diagnosis | https://www.improvediagnosis.org/dxiq-column/ask-these-key-questions-when-you-get-a-diagnosis/ |
| G140 | Esposito L, et al. | 2021 | US News | 17 Questions Doctors Wish Their Patients Would Ask | https://health.usnews.com/conditions/slideshows/questions-doctors-wish-their-patients-would-ask |
| G054 | Fallabel C. | 2022 | Diabetes Strong | 10 Questions to Ask Your Endocrinologist | https://diabetesstrong.com/questions-to-ask-your-endocrinologist/ |
| G266 | Falson S. | 2020 | WebMD® | Deciding on Your Cancer Treatment: Questions to Ask Your Doctor | https://www.WebMD®.com/cancer/deciding-on-your-cancer-treatment-questions-to-ask-your-doctor |
| G253 | Felson S. | 2020 | WebMD® | Questions to Ask Your Doctor About HIV/AIDS | https://www.WebMD®.com/hiv-aids/questions-doctor-aids |
| G254 | Felson S. | 2021 | WebMD® | Questions to Ask Your Doctor About Ovarian Cancer | https://www.WebMD®.com/ovarian-cancer/guide/questions-doctor-ovarian-cancer |
| G175 | Foltz-Gray D. | 2013 | AARP® | 10 Questions Women Over 50 Should Ask Their Doctor | https://www.AARP®.org/health/conditions-treatments/info-02-2013/questions-women-should-ask-doctors.html |
| G176 | Foltz-Gray D. | Mar | AARP® | 10 Questions Every Man Over 50 Should Ask His Doctor | https://www.AARP®.org/health/conditions-treatments/info-03-2013/questions-men-should-ask-doctors.html |
| G221 | Funk K. | 2010 | TODAY | Diagnosed with breast cancer? 8 things to ask the doctor | https://www.today.com/health/diagnosed-breast-cancer-8-things-ask-doctor-2D80555416 |
| G022 | Ghauri M. | 2020 | Spine & Pain Clinics of North America | What Questions to Ask a Pain Management Doctor? | https://www.sapnamed.com/blog/what-questions-to-ask-a-pain-management-doctor/ |
| P093 | Hamel LM, et al. | 2021;22(1):636 | Trials | DISCO App: study protocol for a randomized controlled trial to test the effectiveness of a patient intervention to reduce the financial burden of cancer in a diverse patient population | 10.1186/s13063-021-05593-y |
| G237 | Heerema E. | 2022 | VeryWell Health | 12 Things to Ask the Healthcare Provider After a Dementia Diagnosis | https://www.verywellhealth.com/dementia-diagnosis-questions-98857 |
| G224 | Heid M. | 2016 | TIME | 9 Questions Your Doctor Wishes You'd Ask | https://time.com/4433153/9-questions-ask-doctor/ |
| G250 | Hoffman M, et al. | 2022 | WebMD® | Hyperhidrosis: 10 Questions to Ask Your Doctor | https://www.WebMD®.com/skin-problems-and-treatments/hyperhidrosis-10-questions-to-ask-your-doctor |
| G251 | Hoffman M, et al. | 2021 | WebMD® | Questions to Ask Your Doctor About Psoriasis Treatments | https://www.WebMD®.com/skin-problems-and-treatments/psoriasis/questions |
| G048 | Howley EK. | 2018 | US News | 6 Questions to Ask Your Doctor When You’re Diagnosed With Diabetes | https://health.usnews.com/health-care/patient-advice/articles/2018-06-22/6-questions-to-ask-your-doctor-when-youre-diagnosed-with-diabetes |
| G252 | Jaliman D. | 2021 | WebMD® | 12 Questions to Ask Your Doctor About Melanoma | https://www.WebMD®.com/melanoma-skin-cancer/questions-doctor-melanoma |
| P059 | Jayasekera J, et al. | 2020;16(10):e1085-e1097 | JCO Oncol Pract | Question prompt list to support patient-provider communication in the use of the 21-gene recurrence test: Feasibility, acceptability, and outcomes | 10.1200/JOP.19.00661 |
| P081 | Jenkins HR, et al. | 2022;28(6):896-905 | J Card Fail | Examining information needs of heart failure patients and family companions using a previsit question prompt list and audiotaped data: Findings from a pilot study | 10.1016/j.cardfail.2021.11.012 |
| G249 | Johnson T. | 2021 | WebMD® | Questions to Ask Your Doctor About Skin Allergies | https://www.WebMD®.com/allergies/skin-allergy-questions-doctor |
| G274 | Johnson T. | 2022 | WebMD® | Endometriosis | https://www.WebMD®.com/women/endometriosis/endometriosis-causes-symptoms-treatment |
| G276 | Johnson T. | 2020 | WebMD® | What to Ask Your Doctor About Genital Herpes | https://www.WebMD®.com/genital-herpes/genital-questions-doctor |
| G041 | Johnson TC. | 2021 | WebMD® | 10 Osteoporosis Questions to Ask Your Doctor | https://www.WebMD®.com/osteoporosis/guide/10-questions-to-ask-your-doctor-about-osteoporosis |
| P071 | Khan NN, et al. | 2018;110(3):514-522 | Fertil Steril | Development of a question prompt list for women with polycystic ovary syndrome | 10.1016/j.fertnstert.2018.04.028 |
| G247 | Khatri M. | 2021 | WebMD® | 10 Questions to Ask Your Doctor About Kidney Failure | https://www.WebMD®.com/a-to-z-guides/10-important-questions-ask-your-doctor-about-kidney-failure |
| P020 | Kim GS, et al. | 2019;30(5):575-583 | J Assoc Nurses AIDS Care | Development of a question prompt list for patients living with HIV and assessment of their information needs | 10.1097/JNC.0000000000000080 |
| P029 | Lambert K, et al. | 2019;20(1):48 | BMC Nephrol | Development and preliminary results on the feasibility of a renal diet specific question prompt sheet for use in nephrology clinics | 10.1186/s12882-019-1231-3 |
| G233 | Leader D. | 2022 | VeryWell Health | 10 Questions to Ask Your Healthcare Provider About COPD | https://www.verywellhealth.com/questions-for-your-doctor-copd-914753 |
| P315 | Lederer S, et al. | 2016;17(1):155 | BMC Nephrol | A question prompt sheet for adult patients with chronic kidney disease | 10.1186/s12882-016-0362-z |
| P039 | Lemmon ME, et al. | 2019;34(11):653-659 | J Child Neurol | Neurodevelopmental risk: A tool to enhance conversations with families of infants | 10.1177/0883073819844927 |
| G004 | Lim S, et al. | 2021 | Oak St. Health | 100+ Important Questions to Ask a Doctor | https://www.oakstreethealth.com/100-important-questions-to-ask-a-doctor-528298 |
| P005 | Low JTS, et al. | 2020;54(10):898-904 | J Clin Gastroenterol | Improving communication in outpatient consultations in people with cirrhosis: The development of a question prompt list (QPL) | 10.1097/MCG.0000000000001347 |
| P325 | Mancini J, et al. | 2015;39(6):599-605 | Leuk Res | Question prompt list responds to information needs of myelodysplastic syndromes patients and caregivers | 10.1016/j.leukres.2015.03.011 |
| P004 | Mariano DJ, et al. | 2021;479(2):225-232 | Clin Orthop Relat Res | Does a question prompt list improve perceived involvement in care in orthopaedic surgery compared with the AskShareKnow questions? A pragmatic randomized controlled trial | 10.1097/CORR.0000000000001582 |
| G235 | Martin C. | 2022 | VeryWell Health | Questions to Ask Before Starting a New MS Medication | https://www.verywellhealth.com/ms-medication-checklist-for-your-next-appointment-5536935 |
| G277 | Martin LJ. | 2020 | WebMD® | What should I ask my doctor about HER2-positive breast cancer? | https://www.WebMD®.com/breast-cancer/qa/what-are-questions-for-your-doctor-regarding-her2positive-breast-cancer |
| P373 | Matsuoka A, et al. | 2022;12(9):e063445 | BMJ Open | Geriatric assessment and management with question prompt list using a web-based application for elderly patients with cancer (MAPLE) to communicate ageing-related concerns: J-SUPPORT 2101 study protocol for a multicentre, parallel group, randomised controlled trial | 10.1136/bmjopen-2022-063445 |
| P371 | McDarby M, et al. | 2022;S0885-3924(22)01039-9 | J Pain Symptom Manage | Effects of a Patient Question Prompt List on Question Asking and Self-Efficacy During Outpatient Palliative Care Appointments | 10.1016/j.jpainsymman.2022.12.010 |
| G055 | Melinosky C. | 2020 | WebMD® | 10 Questions to Ask Your Doctor About Parkinson's Disease | https://www.WebMD®.com/parkinsons-disease/questions-doctor-parkinsons |
| G259 | Melinosky C. | 2020 | WebMD® | 10 Questions to Ask Your Doctor About Parkinson's Disease | https://www.WebMD®.com/parkinsons-disease/questions-doctor-parkinsons |
| G260 | Melinosky C. | 2020 | WebMD® | 10 Questions to Ask Your Doctor About Alzheimer's Disease | https://www.WebMD®.com/alzheimers/guide/questions-doctor-alzheimers |
| G059 | Meyer ML. | 2019 | Vital Record, Texas A&M Health | 9 QUESTIONS TO ASK YOUR DOCTOR | https://vitalrecord.tamhsc.edu/9-questions-to-ask-your-doctor/ |
| G243 | Miller K, et al. | 2017 | WebMD® | Questions for Your Doctor About Your Hypothyroidism | https://www.WebMD®.com/women/manage-hypothyroidism-17/effects/low-thyroid-doctor-questions |
| G089 | Nall R, et al. | 2022 | SELF | 7 Questions to Ask Your Doctor After Being Diagnosed With Hidradenitis Suppurativa | https://www.self.com/story/hidradenitis-suppurativa-doctor-questions |
| G262 | Nazario B. | 2020 | WebMD® | What questions should you ask your doctor about polycythemia vera? | https://www.WebMD®.com/cancer/qa/what-questions-should-you-ask-your-doctor-about-polycythemia-vera |
| G279 | Nazario B. | 2022 | WebMD® | What to Ask Your Doctor | https://www.WebMD®.com/ds/ddg-chronic-heart-failure |
| G084 | Nudson R. | 2019 | Elemental | The Smartest Questions to Ask Your Doctor | https://elemental.medium.com/the-smartest-questions-to-ask-your-doctor-b12757820524 |
| G083 | Oglethorpe A. | 2021 | Good Housekeeping | 6 Heart Health Questions to Ask Your Doctor | https://www.goodhousekeeping.com/health/a36958317/heart-health-questions-for-doctor/ |
| G269 | Pagano T. | 2020 | WebMD® | WebMD®'s 10 Important Questions to Ask Your Doctor About Pelvic Pain | https://www.WebMD®.com/women/pelvic-pain-questions |
| G239 | Pathak N. | 2022 | WebMD® | Questions to Ask Your Doctor | https://www.WebMD®.com/sleep-disorders/dr-questions-idiopathic-hypersomnia |
| G245 | Pathak N. | 2021 | WebMD® | Ulcerative Colitis: Questions to Ask Your Doctor | https://www.WebMD®.com/ibd-crohns-disease/ulcerative-colitis/ulcerative-colitis-talk-to-your-doctor |
| G256 | Pathak N. | 2019 | WebMD® | Questions to Ask Your Doctor About PAH | https://www.WebMD®.com/lung/pah-help-16/pulmonary-arterial-hypertension-dr-questions |
| G056 | Patino E, et al. | 2022 | Everyday Health | 5 Questions to Ask Your Doctor About Genomic Testing for NSCLC | https://www.everydayhealth.com/lung-cancer/genomic-testing-can-lead-to-better-treatment-for-non-small-cell-lung-cancer/ |
| G057 | Pearl R. | 2021 | Next Avenue | 5 Key Questions to Ask Your Doctor | https://www.nextavenue.org/questions-ask-doctor/ |
| G023 | Petrini ME. | 2022 | KidsHealth | Questions You Can Ask Your Doctor (for Teens) | https://kidshealth.org/en/teens/questions-doctor.html |
| P008 | Renovanz M, et al. | 2019;19(3):523-531 | Spine J | Information needs of patients in spine surgery: Development of a question prompt list to guide informed consent consultations | 10.1016/j.spinee.2018.08.015 |
| G261 | Robinson J. | 2020 | WebMD® | Questions to Ask Your Doctor About Advanced Breast Cancer | https://www.WebMD®.com/breast-cancer/guide/doc-questions |
| G264 | Robinson J. | 2021 | WebMD® | Questions to Ask Your Doctor About Migraines | https://www.WebMD®.com/migraines-headaches/guide/questions-doctor-migraines |
| G278 | Robinson J. | 2020 | WebMD® | 10 Questions to Ask Doctor About GERD | https://www.WebMD®.com/heartburn-gerd/10-questions-to-ask-doctor-heartburn |
| P088 | Roe AK, et al. | 2021;46(9):818.e1-818.e6 | J Hand Surg Am | Engaging patients to ask more questions: what's the best way? A pragmatic randomized controlled trial | 10.1016/j.jhsa.2021.02.001 |
| G192 | Rossheim J, et al. | 2021 | WebMD® | How to Get Your Doctor to Speak Your Language | https://www.WebMD®.com/a-to-z-guides/features/doctor-patient-miscommunication |
| G271 | Sachdev P. | 2021 | WebMD® | Questions You Should Ask at Every Doctor's Visit | https://www.WebMD®.com/a-to-z-guides/features/cm/5-questions-you-should-ask-at-every-doctors-visit |
| P060 | Schwarze ML, et al. | 2020;155(1):6-13. | JAMA Surg | Effectiveness of a question prompt list intervention for older patients considering major surgery: A multisite randomized clinical trial | 10.1001/jamasurg.2019.3778 |
| G100 | Segal B. | 2020 | Wexner Medical Center, Ohio State University | 5 questions to ask your MS doctor | https://wexnermedical.osu.edu/blog/5-questions-you-should-ask-your-ms-doctor |
| G035 | Sentkoski H. | N/A | Johns Hopkins Medicine, Pathology | What Questions Should I Ask My Doctor? | https://pathology.jhu.edu/pancreas/questions-to-ask |
| P073 | Sleath B, et al. | 2020;57(9):1029-1038 | J Asthma | The impact of a question prompt list and video intervention on teen asthma control and quality-of-life one year later: Results of a randomized trial | 10.1080/02770903.2019.1633542 |
| P377 | Sleath B, et al. | 2022;99(12):838-843 | Optom Vis Sci | Acceptance of a Pre-visit Intervention to Engage African American Glaucoma Patients during Visits | 10.1097/OPX.0000000000001959 |
| G241 | Smith M. | 2021 | WebMD® | Health Guide & Infographic | https://www.WebMD®.com/lung-cancer/early-nsclc-21/early-nsclc-dr-questions |
| G096 | Stoppler MC. | N/A | MedicineNet | Top 10 Questions to Asl Your Doctor About Diabetes | https://www.medicinenet.com/diabetes_questions_to_ask_your_doctor/views.htm |
| P372 | Svensberg K, et al. | 2022;18(12):4072-4082 | Res Social Adm Pharm | Making medication communication visible in community pharmacies-pharmacists' experience using a question prompt list in the patient meeting | 10.1016/j.sapharm.2022.07.011 |
| G231 | Thomas C. | 2021 | Heart Sisters | Six rules for navigating your next doctor’s appointment | https://myheartsisters.org/2012/02/21/six-rules-doctors-appointment/ |
| G265 | Todd N. | 2020 | WebMD® | Questions to Ask Your Doctor About Pelvic Inflammatory Disease | https://www.WebMD®.com/women/questions-doctor-pelvic-inflammatory-disease |
| G236 | Tresca AJ. | 2020 | VeryWell Health | IBD Questions You're Afraid to Ask Your Gastroenterologist | https://www.verywellhealth.com/ibd-questions-youre-too-afraid-to-ask-your-gastro-1942720 |
| P070 | van der Steen JT, et al. | 2021;11(4):e044591 | BMJ Open | Practitioners' perceptions of acceptability of a question prompt list about palliative care for advance care planning with people living with dementia and their family caregivers: A mixed-methods evaluation study | 10.1136/bmjopen-2020-044591 |
| P378 | Verhoef MJ, et al. | 2022;31(6):e13708 | Eur J Cancer Care (Engl) | Assessment of patient symptom burden and information needs helps tailoring palliative care consultations: An observational study | 10.1111/ecc.13708 |
| G040 | Whitlock J. | 2021 | Verywell Health | Important Questions to Ask Before a Hysterectomy | https://www.verywellhealth.com/questions-to-ask-your-surgeon-before-a-hysterectomy-3156956 |
| G118 | Wilson JM. | 2019 | Health Central | 10 Questions to Ask Your Doctor About Ulcerative Colitis | https://www.healthcentral.com/slideshow/questions-to-ask-doctor-about-ulcerative-colitis |
| G133 | Woolston C. | 2020 | HealthDay | Questions to Ask Your Doctor: Diabetes | https://consumer.healthday.com/encyclopedia/diabetes-13/misc-diabetes-news-181/questions-to-ask-your-doctor-diabetes-644932.html |
| G272 | Zelman D. | 2020 | WebMD® | Questions to Ask Your Doctor About Fibromyalgia and Myalgic Encephalomyelitis/Chronic Fatigue Syndrome | https://www.WebMD®.com/fibromyalgia/questions-doctor-fibromyalgia-cfids |
| G002 | N/A | N/A | Cleveland Clinic | Questions to Ask Your Doctor | https://my.clevelandclinic.org/patients/information/questions-to-ask-your-doctor |
| G003 | N/A | 2020 | National Institutes of Aging (NIA) | What Should I Ask My Doctor During a Checkup? | https://www.nia.nih.gov/health/what-should-i-ask-my-doctor-during-checkup |
| G007 | N/A | 2020 | Conway Medical Center | The Top 10 Questions You Should Ask Your Primary Care Provider | https://www.conwaymedicalcenter.com/news/the-top-10-questions-you-should-ask-your-primary-care-doctor/ |
| G011 | N/A | 2017 | Elder Care Alliance | What’s Up, Doc? Questions to Ask Your Parent’s Doctor | https://eldercarealliance.org/blog/whats-up-doc-questions-to-ask-your-parents-doctor/ |
| G014 | N/A | N/A | MedicineNet | Questions to Ask Your Doctor | https://www.medicinenet.com/questions_to_ask_your_doctor_-_general/views.htm |
| G015 | N/A | N/A | Understand Cancer Together | Questions to ask your doctor | https://www.understandcancertogether.com/questions-to-ask-your-doctor/ |
| G018 | N/A | 2010 | Hospital for Special Surgery | Questions to Ask Your Doctor About Your Condition | https://www.hss.edu/conditions_questions-to-ask-doctor.asp |
| G019 | N/A | N/A | Parkinson's Foundation | Key Questions For Your Doctor’s Visit | https://www.parkinson.org/pd-library/tip-sheets/questions-for-doctors-visit |
| G020 | N/A | 2008 | American Psychological Association | Questions to ask your doctor | https://www.apa.org/topics/behavioral-health/questions-doctor |
| G024 | N/A | 2015 | Rush University Medical Center | Questions to Ask Your Doctor | https://www.rush.edu/news/questions-ask-your-doctor |
| G025 | N/A | N/A | HealthLink | What to ask your doctor | https://www.healthlink.com/documents/questions_to_ask_your_doctor.pdf |
| G027 | N/A | 2021 | American Cancer Society® | Questions to Ask About Your Cancer | https://www.cancer.org/content/dam/cancer-org/cancer-control/en/worksheets/questions-to-ask-about-my-cancer.pdf |
| G033 | N/A | 2022 | American Heart Association® | Heart Failure: Partnering in Your Treatment | https://www.heart.org/-/media/Files/Health-Topics/Heart-Failure/Heart-Failure-Partnering-in-Your-Treatment.pdf |
| G034 | N/A | 2017 | American Heart Association® | Heart Failure Questions to Ask Your Doctor | https://www.heart.org/en/health-topics/heart-failure/heart-failure-tools-resources/heart-failure-questions-to-ask-your-doctor |
| G037 | N/A | 2018 | City of Hope® | Seven questions you should ask your doctor before getting a colonoscopy | https://www.cancercenter.com/community/blog/2018/03/seven-questions-you-should-ask-your-doctor-before-getting-a-colonoscopy |
| G046 | N/A | N/A | Indiana University, HANDS in Autism | Questions to Ask Your Doctor | https://handsinautism.iupui.edu/about-autism/questions-to-ask-your-doctor/ |
| G047 | N/A | 2022 | Healthline | 8 Questions to Ask Your Doctor About Schizophrenia | https://www.healthline.com/health/schizophrenia/what-to-ask-doctor-about-schizophrenia |
| G049 | N/A | 2022 | We Can Do This | Questions to Ask Your Doctor About COVID-19 and Your Chronic Illness | https://wecandothis.hhs.gov/resource/oa-questions-to-ask-your-doctor-about-covid-19-and-your-chronic-illness |
| G051 | N/A | 2018 | Nontuberculous Mycobacteria (NTM) Info and Research Inc. | Questions to Ask Your Doctor | https://ntminfo.org/questions-to-ask-your-doctor/ |
| G060 | N/A | 2016 | American Heart Association® | Questions to Ask Your Doctor: Implantable Cardioverter Defibrillator (ICD) | https://www.heart.org/en/health-topics/arrhythmia/prevention--treatment-of-arrhythmia/questions-to-ask-your-doctor--implantable-cardioverter-defibrillator-icd |
| G061 | N/A | 2021 | American Heart Association® | Working with Your Diabetes Health Care Team | https://www.heart.org/en/health-topics/diabetes/prevention--treatment-of-diabetes/work-with-your-health-care-team |
| G062 | N/A | 2019 | American Heart Association® | 4 Questions to Ask Your Doctor About Diabetes and Your Heart | https://www.knowdiabetesbyheart.org/wp-content/uploads/2019/06/KDBH_4QuestionstoAskYourDoc.pdf |
| G063 | N/A | 2018 | American Heart Association® | Cardiac Rehab Questions for Your Healthcare Professional | https://www.heart.org/en/health-topics/cardiac-rehab/communicating-with-professionals/cardiac-rehab-questions-for-your-healthcare-professional |
| G066 | N/A | 2019 | NHS (UK) | What to ask your doctor: Checklist of questions to ask at your appointment | https://www.nhs.uk/nhs-services/gps/what-to-ask-your-doctor/ |
| G068 | N/A | 2020 | City of Hope® | 10 questions to ask your doctor during your first visit | https://www.cancercenter.com/community/blog/2020/03/questions-to-ask-doctor-initial-visit-second-opinion |
| G075 | N/A | 2022 | Susan G Komen | Questions You May Want to Ask Your Health Care Provider – Breast Cancer Risk | https://www.komen.org/breast-cancer/risk-factor/questions-for-your-doctor/ |
| G076 | N/A | 2019 | Get Palliative Care | Five Questions to Ask Your Doctor at Diagnosis | https://getpalliativecare.org/five-questions-to-ask-your-doctor-at-diagnosis/ |
| G079 | N/A | 2021 | CardioSmart®, American College of Cardiology® | Questions to Ask | https://www.cardiosmart.org/topics/aortic-aneurysm/questions-to-ask |
| G082 | N/A | 2021 | American Lung Association® | Questions to Ask Your Doctor about COPD | https://www.lung.org/lung-health-diseases/lung-disease-lookup/copd/top-5-questions-to-ask-your-doctor |
| G086 | N/A | 2016 | Chemotherapy.com | Questions to Ask Your Doctor | https://www.chemotherapy.com/new_to_chemo/doctor_and_care_team/questions/ |
| G098 | N/A | 2020 | American Lung Association® | Questions to Ask Your Doctor About Bronchiectasis | https://www.lung.org/lung-health-diseases/lung-disease-lookup/bronchiectasis/questions-to-ask-your-doctor |
| G099 | N/A | N/A | National Jewish Health | Questions To Ask Your Doctor | https://www.nationaljewish.org/patients-visitors/patient-info/prepare-for-your-appointment/ask-your-doctor |
| G103 | N/A | N/A | UCSF Health | Questions to Ask Your Doctor | https://www.ucsfhealth.org/education/questions-to-ask-your-doctor |
| G104 | N/A | 2021 | American Heart Association; American Diabetes Association | Tips for Talking to Your Doctor about Kidney Health | https://www.knowdiabetesbyheart.org/wp-content/uploads/2022/04/Kidney-Health-Discussion-Tips.pdf |
| G108 | N/A | 2022 | Epsy Health (UK) | 15 seizure questions to ask your neurologist | https://www.epsyhealth.com/seizure-epilepsy-blog/15-seizure-questions-to-ask-your-neurologist |
| G110 | N/A | N/A | Hydrocephalus Association | What to Ask Your Doctor About Infants and Children with Hydrocephalus | https://www.hydroassoc.org/questions-for-parents-of-infants-and-children/ |
| G111 | N/A | N/A | Hydrocephalus Association | What to Ask Your Doctor about Teens with Hydrocephalus | https://www.hydroassoc.org/questions-for-teens-and-young-adults/ |
| G112 | N/A | N/A | Hydrocephalus Association | What to Ask Your Doctor about Young and Middle-Age Adults with Hydrocephalus | https://www.hydroassoc.org/questions-for-young-and-middle-age-adults/ |
| G113 | N/A | N/A | Hydrocephalus Association | What to Ask Your Doctor about NPH | https://www.hydroassoc.org/questions-for-adults-with-nph/ |
| G114 | N/A | N/A | British Liver Trust (UK) | Questions to ask your specialist if you have been diagnosed with liver cancer | https://britishlivertrust.org.uk/wp-content/uploads/Qs-to-ask-if-you-have-liver-cancer.pdf |
| G115 | N/A | N/A | British Liver Trust (UK) | Questions to ask your specialist if you have been diagnosed with liver disease | https://britishlivertrust.org.uk/wp-content/uploads/Qs-to-ask-after-a-diagnosis.pdf |
| G116 | N/A | N/A | British Liver Trust (UK) | Questions to ask your specialist if you have been diagnosed with any type of cirrhosis | https://britishlivertrust.org.uk/wp-content/uploads/Qs-to-ask-if-you-have-cirrhosis.pdf |
| G120 | N/A | 2018 | Urology Care Foundation | Prostate Cancer: Questions to Ask your Doctor | https://www.urologyhealth.org/documents/Product-Store/English/Prostate-Cancer-Diagnosis-Questions-to-Ask-Updated-Fact-Sheet.pdf |
| G121 | N/A | 2018 | Know Your Girls | Questions to ask your doctor about breast health | https://knowyourgirls.org/wp-content/uploads/2018/04/Questions-to-ask-your-doctor-about-breast-health.pdf |
| G122 | N/A | N/A | Journey Care | Living With Serious Illness: Questions to Ask Your Doctor | https://journeycare.org/questions-ask-doctor/ |
| G127 | N/A | N/A | University of Maryland Medical Center | 20 Questions to Ask Your Doctor About IBD | https://www.umms.org/ummc/health-services/digestive/conditions/inflammatory-bowel-disease/patient-information/20-questions-ask-your-doctor |
| G128 | N/A | 2017 | PsychCentral | Questions to Ask Your Doctor About Medications | https://psychcentral.com/lib/questions-for-your-doctor-about-medications#1 |
| G131 | N/A | 2017 | Best Doctor | 10 Questions to Ask Your Cardiologist After a Heart Attack | https://bestdoctor.com/blog/questions-to-ask-your-cardiologist-after-a-heart-attack/ |
| G138 | N/A | 2022 | Stop The Clot, National Blood Clot Alliance | Questions to ask your doctor after having a blood clot | https://www.stoptheclot.org/peer-support/questions-to-ask-your-doctor-about-blood-clots/ |
| G141 | N/A | N/A | The Health Literacy Place | Encouraging patient questions | https://www.healthliteracyplace.org.uk/toolkit/encouraging-patient-questions/ |
| G148 | N/A | 2007 | Agency for Healthcare Research and Quality (US) | Do You Know the Right Questions to Ask? | https://www.ahrq.gov/questions/resources/poster.html |
| G149 | N/A | 2020 | Agency for Healthcare Research and Quality (US) | 20 Tips To Help Prevent Medical Errors: Patient Fact Sheet | https://www.ahrq.gov/questions/resources/20-tips.html |
| G150 | N/A | 2020 | Agency for Healthcare Research and Quality (US) | Next Steps After Your Diagnosis | https://www.ahrq.gov/sites/default/files/publications2/files/diaginfo.pdf |
| G154 | N/A | 2020 | Agency for Healthcare Research and Quality (US) | The 10 Questions You Should Know | https://www.ahrq.gov/questions/10questions.html |
| G162 | N/A | 2020 | Agency for Healthcare Research and Quality (US) | Be More Engaged in Your Healthcare- Tips for Patients | https://www.ahrq.gov/sites/default/files/wysiwyg/questions/be-engaged.pdf |
| G164 | N/A | N/A | RT Answers | Questions To Ask Before Treatment | https://www.rtanswers.org/What-is-Radiation-Therapy/Questions-To-Ask-Your-Doctor/Questions-To-Ask-Before-Treatment |
| G165 | N/A | N/A | RT Answers | Questions to Ask During Treatment | https://www.rtanswers.org/RTAnswers/media/RTAnswers/What%20is%20radiation%20therapy/PDFs/RTAnswer_QuestionToAsk_DuringTreatment.pdf |
| G166 | N/A | N/A | RT Answers | Questions To Ask After Treatment Ends | https://www.rtanswers.org/RTAnswers/media/RTAnswers/What%20is%20radiation%20therapy/PDFs/RTAnswer_QuestionToAsk_AfterTreatment.pdf |
| G167 | N/A | N/A | RT Answers | Questions To Ask About Radiation Safety | https://www.rtanswers.org/RTAnswers/media/RTAnswers/What%20is%20radiation%20therapy/PDFs/RTAnswer_QuestionToAsk_RadiationSafety.pdf |
| G168 | N/A | 2022 | Agency for Healthcare Research and Quality (US) | Four Questions To Ask Your Doctor or Nurse About Antibiotics | https://www.ahrq.gov/sites/default/files/wysiwyg/antibiotic-use/long-term-care/four-moments-residents.pdf |
| G169 | N/A | 2017 | Agency for Healthcare Research and Quality (US) | Be Prepared to Be Engaged- Patient Note Sheet | https://www.ahrq.gov/sites/default/files/wysiwyg/professionals/quality-patient-safety/patient-family-engagement/pfeprimarycare/bepreparedptnotesheet.pdf |
| G170 | N/A | 2018 | SIDM | The Patient’s Toolkit for Diagnosis | https://www.improvediagnosis.org/wp-content/uploads/2018/10/Patient_Toolkit_-_Fillable.pdf |
| G171 | N/A | 2020 | Joint Commission | Speak Up: Tips for your doctor’s visit | https://www.jointcommission.org/-/media/tjc/documents/resources/speak-up/doctors_visit_brochure-5-15-2020.pdf?db=web&hash=5122FF476AE7D67E72A0FB809AE3E94E&hash=5122FF476AE7D67E72A0FB809AE3E94E |
| G172 | N/A | 2020 | Joint Commission | Speak Up: Help prevent medical test mistakes | https://www.jointcommission.org/-/media/tjc/documents/resources/speak-up/speakup-medical-tests-5-15-2020.pdf?db=web&hash=9A1B292C6892ABB6DB5288ECCE3A51B8&hash=9A1B292C6892ABB6DB5288ECCE3A51B8 |
| G173 | N/A | 2010 | AARP® | Questions Caregivers Need to Ask at Doctor Appointments | https://www.AARP®.org/caregiving/health/info-2017/questions-to-ask-doctor.html |
| G179 | N/A | N/A | CHEST® Foundation, American College of Chest Physicians® | Questions to Ask Your doctor | https://foundation.chestnet.org/wp-content/uploads/2021/05/Questions-to-ask-your-doctor-CHEST-Foundation.pdf |
| G180 | N/A | N/A | American Heart Association® | Health Literacy \| Understanding What Your Doctor Is Saying | https://www.heart.org/en/health-topics/consumer-healthcare/doctor-appointments-questions-to-ask-your-doctor/health-literacy--understanding-what-your-doctor-is-saying |
| G181 | N/A | 2018 | American Heart Association® | Preparing for Medical Visits | https://www.heart.org/en/health-topics/cardiac-rehab/communicating-with-professionals/preparing-for-medical-visits |
| G182 | N/A | 2018 | American Heart Association® | Taking Control of Your Medicines | https://www.heart.org/en/health-topics/cardiac-rehab/managing-your-medicines/taking-control-of-your-medicines |
| G183 | N/A | 2021 | American Heart Association® | Patient/Health Care Provider Discussion Guide | https://www.heart.org/-/media/Files/Health-Topics/Heart-Failure/Heart-Failure-Discussion-Guide.pdf |
| G184 | N/A | 2021 | Centers for Disease Control and Prevention (US) | Monitoring Your Blood Sugar | https://www.cdc.gov/diabetes/managing/managing-blood-sugar/bloodglucosemonitoring.html |
| G185 | N/A | 2022 | National Cancer Institute | Questions to Ask Your Doctor about Your Diagnosis | https://www.cancer.gov/about-cancer/diagnosis-staging/questions |
| G186 | N/A | 2022 | National Cancer Institute | Questions to Ask Your Doctor about Your Treatment | https://www.cancer.gov/about-cancer/treatment/questions |
| G188 | N/A | N/A | Centers for Disease Control and Prevention (US) | Managing Your Diabetes: 5 Questions to Ask Your Health Care Team | https://www.cdc.gov/diabetes/pdfs/managing/5-questions-h.pdf |
| G189 | N/A | 2020 | Centers for Medicare & Medicaid Services (US) | Prepare for a Behavioral Health Appointment | https://www.cms.gov/outreach-education/american-indianalaska-native/behavioral-health/prepare-behavioral-health-appointment |
| G190 | N/A | 2018 | Choosing Wisely®, American Board of Internal Medicine® | 5 QUESTIONS to Ask Your Doctor Before You Get Any Test, Treatment, or Procedure | https://www.choosingwisely.org/wp-content/uploads/2018/03/5-Questions-Poster_18x24-EngLL.pdf |
| G193 | N/A | N/A | Shared Health Manitoba | It's Safe to Ask Brochures | https://sharedhealthmb.ca/patient-care/quality-patient-safety-learning/patient-safety/its-safe-to-ask/ |
| G194 | N/A | 2022 | HRSA | About Transplantation | https://bloodstemcell.hrsa.gov/transplant-basics/about-transplantation |
| G195 | N/A | 2022 | HRSA | Newborn Screening Results and Follow-Up | https://newbornscreening.hrsa.gov/newborn-screening-process/newborn-screening-results-and-follow |
| G196 | N/A | 2021 | OASH | Uterine fibroids | https://www.womenshealth.gov/a-z-topics/uterine-fibroids |
| G197 | N/A | 2020 | NIA, NIH | Discussing Health Decisions with Your Doctor | https://www.nia.nih.gov/health/discussing-health-decisions-your-doctor |
| G198 | N/A | 2018 | American Society of Clinical Oncology® | Questions to Ask Your Health Care Team | https://www.cancer.net/navigating-cancer-care/diagnosing-cancer/questions-ask-your-health-care-team |
| G199 | N/A | 2007 | Agency for Healthcare Research and Quality (US) | Be Prepared for Medical Appointments: Build your question list [Archived] | https://archive.ahrq.gov/qual/beprepared.htm |
| G200 | N/A | 2019 | Joint Commission | Speak Up™ About Your Care | https://www.jointcommission.org/-/media/tjc/documents/resources/speak-up/speak-ups/about-your-care/speak-up-about-your-care-infographic-2019-85x11.pdf |
| G202 | N/A | N/A | American College of Surgeons | 10 Questions to Ask Before Having an Operation | https://www.facs.org/for-medical-professionals/education/for-your-patients/prepare-your-patients-well/10-questions/ |
| G203 | N/A | N/A | AARP® | How to ask the right questions and get the most from your medicines | https://assets.AARP®.org/www.AARP®.org_/articles/health/docs/LetsTalkAboutMeds.pdf |
| G204 | N/A | 2019 | Mayo Clinic | How to make the most of your Mayo Clinic appointment | https://www.mayoclinic.org/patient-visitor-guide/preparing-for-your-visit/how-to-make-the-most-of-your-appointment |
| G205 | N/A | N/A | Canadian Cancer Society | Questions to ask about diagnosis | https://cancer.ca/en/living-with-cancer/coping-with-changes/working-with-your-healthcare-team/questions-to-ask/diagnosis-questions |
| G207 | N/A | 2014 | NIA, NIH | Tips for Talking With Your Doctor | https://www.nia.nih.gov/sites/default/files/d7/talking_with_your_doctor_presentation_handouts_508.pdf |
| G208 | N/A | 2015 | NIH | Talking With Your Doctor: Make the Most of Your Appointment | https://newsinhealth.nih.gov/2015/06/talking-your-doctor |
| G209 | N/A | 2021 | NCI, NIH | Communication in Cancer Care (PDQ®)–Patient Version | https://www.cancer.gov/about-cancer/coping/adjusting-to-cancer/communication-pdq |
| G210 | N/A | N/A | NIDDk, NIH | What questions should I ask my doctor? | https://www.niddk.nih.gov/-/media/5081CA8DAEB5424486D35DD350593EC8.ashx |
| G211 | N/A | 2009 | NHLBI, NIH | The Heart Truth® For African American Women: An Action Plan | https://www.nhlbi.nih.gov/files/docs/public/heart/factsheet-actionplan-aa.pdf |
| G212 | N/A | N/A | NIAAA Treatment Navigator, NIH | Q&As For Doctors With Addiction Specialties | https://alcoholtreatment.niaaa.nih.gov/how-to-find-alcohol-treatment/how-to-search-what-to-ask/questions-for-addiction-doctors |
| G213 | N/A | 2015 | NIAID, NIH | Talking to Your Doctor About Primary Immune Deficiency Diseases | https://www.niaid.nih.gov/diseases-conditions/pidds-talking-your-doctor |
| G214 | N/A | 2014 | NIA, NiH | Talking With Your Doctor: Taking an active role in your healthcare | https://view.officeapps.live.com/op/view.aspx?src=https%3A%2F%2Fwww.nia.nih.gov%2Fsites%2Fdefault%2Ffiles%2Fd7%2Ftalking_with_your_doctor_presentation_powerpoint-508.pptx&wdOrigin=BROWSELINK |
| G215 | N/A | 2022 | NCI, NIH | Questions to Ask Your Doctor When You Have Finished Treatment | https://www.cancer.gov/about-cancer/coping/survivorship/questions |
| G216 | N/A | 2022 | NCI, NIH | Questions to Ask Your Doctor about Advanced Cancer | https://www.cancer.gov/about-cancer/advanced-cancer/questions |
| G217 | N/A | N/A | NHLBI, NIH | Questions To Ask The Doctor | https://www.nhlbi.nih.gov/sites/default/files/publications/QuestionsToAskTheDoctor.pdf |
| G218 | N/A | 2021 | NEI, NIH | Talking with Your Eye Doctor | https://www.nei.nih.gov/learn-about-eye-health/healthy-vision/finding-eye-doctor/talking-your-eye-doctor |
| G225 | N/A | N/A | Health Direct | Questions to ask your doctor | https://www.healthdirect.gov.au/questions-to-ask-your-doctor |
| G229 | N/A | 2021 | Cancer Council NSW (Australia) | Questions to ask your doctor | https://www.cancercouncil.com.au/wp-content/uploads/2021/07/Questions-to-ask-your-doctor.pdf |
| G230 | N/A | 2018 | BC Injury Research and Prevention Unit (Canada) | QUESTIONS TO ASK YOUR DOCTOR (For Parents) | https://cattonline.com/wp-content/uploads/2017/10/CATT-Questions-to-Ask-Your-Doctor-Parents-V2-January-2018.pdf |
| G232 | N/A | N/A | WomenHeart | Questions for your Doctor | https://www.womenheart.org/your-heart-journey/questions-for-your-doctor/ |
| G283 | N/A | N/A | VeryWell Mind | Doctor Discussion Guide: Managing ADHD | https://files.verywellmind.com/doctor-discussion-guides/ADHD+Doctor+Discussion+Guide.pdf |
| G284 | N/A | N/A | VeryWell Mind | Doctor Discussion Guide: Managing Anorexia Nervosa | https://files.verywellmind.com/doctor-discussion-guides/Anorexia+Nervosa+Doctor+Discussion+Guide.pdf |
| G285 | N/A | N/A | VeryWell Mind | Doctor Discussion Guide: Managing Binge Eating Disorder | https://files.verywellmind.com/doctor-discussion-guides/Binge+Eating+Disorder+Doctor+Discussion+Guide.pdf |
| G286 | N/A | N/A | VeryWell Mind | Doctor Discussion Guide: Managing Bipolar Disorder | https://files.verywellmind.com/doctor-discussion-guides/Bipolar+Disorder+Doctor+Discussion+Guide.pdf |
| G287 | N/A | N/A | VeryWell Mind | Doctor Discussion Guide: Managing Borderline Personality Disorder | https://files.verywellmind.com/doctor-discussion-guides/Borderline+Personality+Disorder+Doctor+Discussion+Guide.pdf |
| G288 | N/A | N/A | VeryWell Mind | Doctor Discussion Guide: Managing Bulimia Nervosa | https://files.verywellmind.com/doctor-discussion-guides/Bulimia+Nervosa+Doctor+Discussion+Guide.pdf |
| G289 | N/A | N/A | VeryWell Mind | Doctor Discussion Guide: Managing Depression | https://files.verywellmind.com/doctor-discussion-guides/Depression+Doctor+Discussion+Guide.pdf |
| G290 | N/A | N/A | VeryWell Mind | Doctor Discussion Guide: Managing Generalized Anxiety Disorder | https://files.verywellmind.com/doctor-discussion-guides/Generalized+Anxiety+Disorder+Doctor+Discussion+Guide.pdf |
| G291 | N/A | N/A | VeryWell Mind | Doctor Discussion Guide: Managing OCD | https://files.verywellmind.com/doctor-discussion-guides/OCD+Doctor+Discussion+Guide.pdf |
| G292 | N/A | N/A | VeryWell Mind | Doctor Discussion Guide: Managing an Opioid Addiction | https://files.verywellmind.com/doctor-discussion-guides/Opioid+Addiction+Doctor+Discussion+Guide.pdf |
| G293 | N/A | N/A | VeryWell Mind | Doctor Discussion Guide: Managing Orthorexia | https://files.verywellmind.com/doctor-discussion-guides/Orthorexia+Doctor+Discussion+Guide.pdf |
| G294 | N/A | N/A | VeryWell Mind | Doctor Discussion Guide: Managing Panic Disorder | https://files.verywellmind.com/doctor-discussion-guides/Panic+Disorder+Doctor+Discussion+Guide.pdf |
| G295 | N/A | N/A | VeryWell Mind | Doctor Discussion Guide: Managing PTSD | https://files.verywellmind.com/doctor-discussion-guides/PTSD+Doctor+Discussion+Guide.pdf |
| G296 | N/A | N/A | VeryWell Mind | Doctor Discussion Guide: Managing Schizophrenia | https://files.verywellmind.com/doctor-discussion-guides/Schizophrenia+Doctor+Discussion+Guide.pdf |
| G297 | N/A | N/A | VeryWell Mind | Doctor Discussion Guide: Managing Social Anxiety Disorder | https://files.verywellmind.com/doctor-discussion-guides/Social+Anxiety+Disorder+Doctor+Discussion+Guide.pdf |
