# Supplementary material for: “What Else Could It Be?” A Scoping Review of Questions for Patients to Ask Throughout the Diagnostic Process
Source: J Patient Saf. Author manuscript; Available in PMC 2025 Feb 7. (PMC11803640; doi:10.1097/PTS.0000000000001273)
Supplement: AppendixA [file NIHMS2048852-supplement-AppendixA.docx]

**Appendix A. Search strategy**

1. Peer Reviewed Literature Searches

**July 12, 2022**

Database: PUBMED <2015 to 2022 July 12>, Google Scholar <2015 to 2022 July 12>, Search strategy:

-------------------------------------------------------------------------------------------

(Question prompt list) AND (consultations)

(Question prompt list) AND (physician encounter)

(Question prompt list) AND (patient encounter)

(Question asking) AND (consultations)

(Question asking) AND (physician encounter)

(Question asking) AND (patient encounter)

(Question prompt sheet)

**February 10, 2023**

Database: PUBMED <2015 to 2022 July 12>, Google Scholar <2015 to 2022 July 12>, Search strategy:

-------------------------------------------------------------------------------------------

(Question prompt list) AND (consultations)

(Question prompt list) AND (physician encounter)

(Question prompt list) AND (patient encounter)

(Question asking) AND (consultations)

(Question asking) AND (physician encounter)

(Question asking) AND (patient encounter)

(Question prompt sheet)

1. Grey Literature Searches

**July - August 2022**

*Databases/Websites Searched*

| **Website Type** | **Website Name** |
| --- | --- |
| Search engine | DuckDuckGo |
| Medical Organizations, Associations, and Societies | American College of Chest Physicians  American College of Physicians  American Heart Association*  American Hospital Association  American Medical Association  American Organization for Nursing Leadership  Children's Hospital Association  Health Research and Educational Trust (HRET)  The National Academies of Science Engineering and Medicine |
| Hospitals and Health Systems | Brigham and Women’s Hospital  Cedars-Sinai  Cleveland Clinic  Johns Hopkins  Mayo Clinic  UCLA Medical Center |
| Patient and Diagnostic Safety Organizations | Australian Commission on Safety and Quality in Health Care  Canadian Patient Safety Institute (now Healthcare Excellence Canada)  Informed Patient Institute  Institute for Healthcare Communication  Institute for Healthcare Improvement  Institute for Patient and Family-Centered Care  National Patient Safety Foundation  Partnership for Patient Safety  Patient Centered Outcomes Research Institute (PCORI)  Society to Improve Diagnosis in Medicine  The Empowered Patient Coalition  World Health Organization – Patients for Patient Safety |
| Government Agencies | Agency for Healthcare Research and Quality (AHRQ)  Centers for Disease Control  Centers for Medicare & Medicaid Services  Department of Defense  DHHS Partnership for Patients  Health Resources and Services Administration  Medicare.gov  National Center for Complementary and Integrative Health  National Institute on Aging  National Institutes for Health |
| Online Health News/Magazine Sources | Modern Healthcare  Medical Xpress  NBC News – Health News  New York Times – Health  STAT News  The Wall Street Journal – Health  US News-Health |
| Other Health-Related Websites and Organizations | AARP  Consumer Med Safety  KidsHealth.org  Medicare Rights Center  Very Well Health  Web MD  World Health Organization* |

*Search Terms and Phrases*

“Questions for patients to ask”

“Questions to ask my doctor”

“Questions to ask in primary care visits”

“What to ask my doctor”

“What to ask in primary care visits”

“Patient questions”*

“Question prompt lists”

“Question asking”*

“Questions”*

“Patient engagement”*

“Patient and family engagement”*

“PFE”*

*****Note: Broad grey literature search terms were only used for targeted website searches when the more specific phrases did not return any results

**February 2023**

*Databases/Websites Searched*

| **Website Type** | **Website Name** |
| --- | --- |
| Search engine | DuckDuckGo |

*Search Terms and Phrases*

“Questions for patients to ask”

“Questions to ask my doctor”

“Questions to ask in primary care visits”

“What to ask my doctor”

“What to ask in primary care visits”

“Patient questions”*

“Question prompt lists”

“Question asking”*

“Questions”*

“Patient engagement”*

“Patient and family engagement”*

“PFE”*
