# Supplementary material for: “What Else Could It Be?” A Scoping Review of Questions for Patients to Ask Throughout the Diagnostic Process
Source: J Patient Saf. Author manuscript; Available in PMC 2025 Feb 7. (PMC11803640; doi:10.1097/PTS.0000000000001273)
Supplement: AppendixB [file NIHMS2048852-supplement-AppendixB.docx]

**Appendix B. Coding Templates**

**Template 1: Publication Citation Information (Resource Level)**

Initial codes for the template analysis involve categorizing articles/resources by their general citation information, including the study/publication type. To this end, we collect descriptive information from each study including: literature source (peer-reviewed or grey literature) title, abstract or description, universal record locator, and unique record identifier. Abstractors then conduct an initial screen of articles/resources for exclusion criteria, to determine whether the article or resource should be included in the full review.

| **Citation Information** | **Operational Definition** |
| --- | --- |
| Resource ID | Resource identification number assigned by the research team. |
| Literature Source | The source of the intervention – was this collected from the peer reviewed or grey literature? |
| Title | Title of the article/resource. |
| Abstract or Description | Article abstract or short description of the article/resource if no abstract is provided. |
| Author | The author(s) of the publication or resource. |
| Journal name/Organization name | Name of the journal or organization where the article/resource was published. |
| Volume | Article/journal volume number (for peer-reviewed articles only). |
| Issue | Article/journal issue number (for peer-reviewed articles only). |
| Page Number(s) | Article/journal page number (for peer-reviewed articles only). |
| Date Published | The date (or year, depending on what is available) of resource/article publication. Date updated or copyright date for grey literature. |
| URL/Web address | URL of the resource website (grey literature) or doi for the journal article (if applicable). |
| Date Accessed | The date (or year, if available) the resource was accessed. |
| Unique Record Identifier | Unique identifier for peer reviewed literature. |
| Exclusion | 1. Not available in English 2. Resource not meant for patients/families 3. Resource does not include at least one question for patients to ask in their appointments 4. Full QPL not available 5. Duplicates |

**Template 2: Secondary Inclusion Screen– First Screen (Resource Level)**

Next, categorize the intended primary end user of the interventions and the specific characteristics of users, if information is available. This includes coding for the demographics of the primary end user (e.g., specific age groups, genders/sexes, races), and/or targeted diagnoses/disease states.

| **Domain** | **Sub-domain(s)** | **Definition: Article/resource is intended for…** |
| --- | --- | --- |
| Condition type | Acute or new conditions (Y/N) | Article/resource is intended for patients with new or acute conditions (e.g., heart attack, fractured limb) |
|  | Chronic conditions (Y/N) | Article/resource is intended for patients with chronic conditions that are being monitored over time for new, improved, or worsened symptoms (e.g., diabetes, chronic kidney disease) |
|  | Specific diagnosis or condition (List) | Article/resource is intended for patients with a specific diagnosis or condition (e.g., heart disease, stroke, cancer) |
|  | Surgical population (Y/N) | Article/resource is intended for patients considering or scheduled for surgery. |
| Demographic of focus | Specific age group (Categories) | Article/resource is intended for one of the following categories: pediatrics (under 18), adults (18-64), older adults (65+) |
|  | Sex group (Categories) | Article/resource is intended for one of the following: females, males, or intersex |
|  | Specific race (Y/N) | Article/resource is intended for a specific racial demographic (e.g., Black or African American, Indigenous persons) |
|  | Other (List) | Other specific populations or groups (List them) |
| Number of questions | Number of questions (#) | What is the number of questions in this article/resource? |
| Exclusion criteria | Not available in English | Article/resource is not available in English |
|  | Not patient-facing | Article/resource was not meant for use by patients, families, and/or caregivers |
|  | No questions | Article/resource does not include at least one question for patients to ask in their appointments |
|  | Outside scope | Resource is not related to diagnosis or is exclusively about pregnancy/fertility |
|  | Not available | Article/resource was unable to be retrieved |
|  | Duplicate | Article/resource is a duplicate |

**Template 3: Intervention Question Domains (Question Level)**

Finally, we code individual questions based on relevant stages of the diagnostic process outlined in the NASEM report. We replace the first step in the diagnostic process (i.e., “Patient experiences a health problem”) with “Pre-engagement.” This is because there may be many factors patients experience that influence their decisions to access the healthcare system, in addition to their experiencing a health problem.

Notes on coding decisions:

1. If you are unsure of how to code something, do your best and then mark it for adjudication. We will discuss these as a team
2. Double coding for diagnostic process steps and overarching themes is acceptable. Categories are not meant to be mutually exclusive.
3. Questions for patients to ask themselves or questions for providers to ask their patients will be excluded as these are not directly related to our research questions.
4. Diagnostic process codes may depend on context. A generic question such as “Where can I learn more information?” may be coded as tests, treatments, or another category, depending on the context in the question prompt list. If you are unsure, mark for adjudication.

**Adjudicate?**

| **Code** | **Definition** |
| --- | --- |
| Mark for adjudication | Mark a question for adjudication if you are unsure about how to code the question. We will discuss these as a team. |

**Diagnostic Process Steps**

| **Questions about…** | **Definition** |
| --- | --- |
| Pre-engagement | Questions for the patient to ask prior to accessing the health care system for a new diagnosis or condition. This may include questions about new appointments, identifying symptoms, or other questions to help patients make decisions about when, how, or where to access care. |
| Patient Engagement in the Health System | Questions for the patient to ask when accessing the health care system. This includes questions related to insurance, cost of care, what/who to bring with you to your appointment, and the care team’s contact information. |
| Clinical History & Interview | Questions for the patient to ask during a discussion of their family history, social history, medical history, and other history of events leading to the health problem. |
| Physical Exam | Questions for the patient to ask related to the routine physical examination. This includes examination of blood pressure, weight, temperature, etc. |
| Diagnostic Testing | Questions for the patient to ask about tests that go beyond the routine physical exam. These tests may include EKGs, CT scans, MRIs, mammograms, colonoscopies, blood tests, stool tests, etc. |
| Referrals and Consultations | Questions for patients to ask about referrals to providers outside of their typical care team. This may include condition-specific specialists (cardiologists, oncologists, etc.), diagnostic specialists (radiologists, pathologists, etc.), or other doctors consulting on the patient’s care (other primary care physicians, internists, dieticians, etc.) |
| Communication of the Diagnosis | Questions for patients to ask about the provider’s explanation of the health problem (diagnosis) or working diagnosis. Includes questions about the patient’s prognosis, alternative diagnoses (differentials), explaining the diagnosis in the patient’s own terms, and where to get more information. |
| Treatment | Questions to help engage patients in shared decision-making around treatment and treatment goals, or decision not to treat. This could include questions about therapies, medicines, surgeries, or other approaches to improve or manage a patient’s condition, including social prescribing. |
| Outcomes | Questions about patient outcomes and post-engagement work after the treatment plan is decided on. Also includes questions to help patients make decisions about when, how, or where to re-engage in care. |

**Exclusion Criteria**

| **Criterion** | **Definition** |
| --- | --- |
| Questions for patients to ask themselves | Questions designed for introspection; can be pre-appointment questions (e.g., to help patients decide whether or not to seek care) or questions designed to help patients communicate during appointments (e.g., questions about their clinical history or symptoms) |
| Questions for providers to ask patients | Questions designed for providers to ask their patients. Could include questions to help gather information from patients, questions about the patient’s clinical history, “teach-back” questions, etc. |
