# Supplementary material for: “What Else Could It Be?” A Scoping Review of Questions for Patients to Ask Throughout the Diagnostic Process
Source: J Patient Saf. Author manuscript; Available in PMC 2025 Feb 7. (PMC11803640; doi:10.1097/PTS.0000000000001273)
Supplement: AppendixD [file NIHMS2048852-supplement-AppendixD.docx]

|  | | **Appendix D: Characteristics of question lists in the peer-reviewed and grey literature** | | | | | | | | |
| --- | --- | --- | --- | --- | --- | --- | --- | --- | --- | --- |
| **ID** | **Number of Questions** | | **Were the questions aimed at individuals with a specific diagnosis or condition type?** | | | | **Were the questions aimed at individuals of a specific demographic?** | | | |
|  | **#** | | **Acute or new condition** | **Existing diagnosis or condition** | **Specific diagnosis/ condition** | **Surgical population** | **Age group** | **Sex** | **Race** | **Other** |
| P003 | 4 | |  | Yes | Cancer |  |  |  |  | Religious or spiritual individuals |
| P004 | 49 | | Yes |  |  | Orthopaedic surgery |  |  |  |  |
| P005 | 22 | | Yes | Yes | Liver cirrhosis |  |  |  |  |  |
| P007 | 112 | |  | Yes | Cancer |  |  |  |  |  |
| P008 | 27 | | Unclear | Unclear |  | Spine surgery |  |  |  |  |
| P011 | 49 | | Yes | Yes | Cancer |  |  |  | Black or African American |  |
| P020 | 12 | | Yes |  | HIV |  |  |  |  |  |
| P023 | 56 | | Yes | Yes | Early stage breast cancer |  |  | Females |  |  |
| P025a | 23 | |  | Yes |  |  | Pediatric |  |  | Family or caregivers |
| P028 | 91 | | Yes |  | Attention-deficit/hyperactivity disorder |  | Pediatric |  |  | Family or caregivers |
| P029 | 18 | |  | Yes | Chronic kidney disease |  |  |  |  |  |
| P039 | 98 | | Yes | Yes | Neurodevelop-mental impairment risk |  | Pediatric |  |  | Family or caregivers |
| P059 | 9 | | Yes |  | Breast cancer |  |  | Females |  |  |
| P060 | 16 | | Unclear | Unclear |  | Any major surgery | Older adults |  |  |  |
| P061 | 55 | | Yes |  | Breast, colon, or lung cancer |  |  |  | Black or African American |  |
| P064 | 20 | |  | Yes | Breast, lung, gastrointestinal, genitourinary and head and neck cancers |  |  |  |  |  |
| P070 | 96 | |  | Yes | Dementia |  | Older adults |  |  |  |
| P071 | 15 | | Yes |  | Polycystic Ovary Syndrome |  |  | Females |  |  |
| P073 | 22 | | Yes |  | Asthma |  | Pediatric |  |  |  |
| P081 | 93 | | Yes | Yes | Heart failure |  |  |  |  | Family or caregivers |
| P088 | 34 | | Yes | Yes |  | Hand surgery |  |  |  |  |
| P093 | 23 | | Yes | Yes | Breast, prostate, lung, or colorectal cancer |  |  |  |  | Economically and racially/ ethnically diverse individuals |
| P187 | 76 | |  | Yes | Heart disease and stroke |  |  |  |  | Patients in cardiac rehab |
| P315 | 32 | | Yes | Yes | Chronic kidney disease |  |  |  |  |  |
| P325 | 53 | | Yes | Yes | Myelodysplastic syndromes |  |  |  |  | Family or caregivers |
| P371 | 25 | |  | Yes |  |  |  |  |  | Family or caregivers |
| P372 | 13 | |  | Yes |  |  |  |  |  | Patients attending community pharmacies |
| P373 | 57 | | Yes |  | Incurable gastrointestinal cancers |  | Older adults |  | Japanese |  |
| P377 | 22 | | Yes |  | Glaucoma |  |  |  | Black or African American |  |
| P378 | 44 | | Yes | Yes |  |  |  |  |  |  |
| G002 | 44 | | Yes | Yes |  |  |  |  |  |  |
| G003 | 19 | | Yes |  |  |  |  |  |  |  |
| G004 | 95 | | Yes |  |  |  |  |  |  |  |
| G007 | 10 | | Yes |  |  |  |  |  |  |  |
| G010 | 36 | | Yes |  | Autoimmune disease |  |  |  |  |  |
| G011 | 17 | | Yes |  |  |  | Older adults |  |  | Family or caregivers |
| G014 | 15 | | Yes |  |  |  |  |  |  |  |
| G015 | 35 | | Yes | Yes | Cancer |  |  |  |  |  |
| G018 | 50 | | Yes |  |  |  |  |  |  |  |
| G019 | 14 | | Yes |  | Parkinson’s |  |  |  |  |  |
| G020 | 14 | |  | Yes |  |  |  |  |  |  |
| G022 | 25 | | Yes |  | Chronic pain |  |  |  |  |  |
| G023 | 24 | | Yes |  |  |  | Pediatric |  |  |  |
| G024 | 26 | | Yes |  |  |  |  |  |  |  |
| G025 | 15 | | Yes |  |  |  |  |  |  |  |
| G027 | 69 | | Yes | Yes | Cancer |  |  |  |  |  |
| G033 | 16 | | Yes |  | Heart failure |  |  |  |  |  |
| G034 | 11 | | Yes |  | Heart failure |  |  |  |  | Family or caregivers |
| G035 | 23 | | Yes |  | Pancreatic cancer |  |  |  |  |  |
| G037 | 7 | | Yes |  |  | Colonoscopy |  |  |  |  |
| G039 | 15 | | Yes | Yes | Multiple sclerosis |  |  |  |  |  |
| G040 | 24 | | Unclear | Unclear |  | Hysterectomy |  | Females |  |  |
| G041 | 10 | | Yes |  | Osteoporosis |  |  |  |  |  |
| G046 | 19 | | Unclear | Unclear | Autism |  | Pediatric |  |  | Family or caregivers |
| G047 | 16 | | Yes |  | Schizophrenia |  |  |  |  |  |
| G048 | 6 | | Yes |  | Diabetes |  |  |  |  |  |
| G049 | 41 | |  | Yes | COVID-19 and chronic illness |  | Older adults |  |  |  |
| G051 | 33 | | Yes |  | Nontuberculous mycobacteria |  |  |  |  |  |
| G054 | 14 | |  | Yes | Diabetes |  |  |  |  |  |
| G055 | 13 | | Yes |  | Parkinson’s |  |  |  |  |  |
| G056 | 5 | | Yes |  | Lung cancer |  |  |  |  |  |
| G057 | 27 | | Unclear | Unclear |  |  |  |  |  |  |
| G059 | 24 | | Yes |  | Dementia |  |  |  |  | Family or caregivers |
| G060 | 21 | | Yes |  |  | Implanting a cardioverter defibrillator |  |  |  | Family or caregivers |
| G061 | 35 | | Yes | Yes | Diabetes |  |  |  |  |  |
| G062 | 4 | | Yes | Yes | Diabetes and heart conditions |  |  |  |  |  |
| G063 | 80 | | Yes | Yes | Heart disease and stroke |  |  |  |  |  |
| G065 | 29 | | Yes |  | Diabetes |  |  |  | Black or African American |  |
| G066 | 22 | | Yes |  |  |  |  |  |  |  |
| G067 | 36 | | Yes | Yes | Lung cancer |  |  |  |  |  |
| G068 | 11 | | Yes |  | Cancer |  |  |  |  |  |
| G075 | 28 | | Yes | Yes | Breast cancer |  |  |  |  |  |
| G076 | 5 | | Yes |  | Any serious illness |  |  |  |  |  |
| G079 | 10 | | Yes |  | Aortic aneurysm |  |  |  |  |  |
| G082 | 5 | |  | Yes | Chronic obstructive pulmonary disease |  |  |  |  |  |
| G083 | 6 | | Yes |  | Heart disease |  |  |  |  |  |
| G084 | 5 | | Yes |  |  |  |  |  |  |  |
| G086 | 25 | | Yes |  | Cancer |  |  |  |  |  |
| G088 | 10 | | Yes |  | Rheumatoid arthritis |  |  |  |  |  |
| G089 | 7 | | Yes |  | Hidradenitis suppurativa |  |  |  |  |  |
| G096 | 14 | | Yes |  | Diabetes |  |  |  |  |  |
| G098 | 11 | | Yes |  | Bronchiectasis |  |  |  |  |  |
| G099 | 37 | | Yes |  |  |  |  |  |  |  |
| G100 | 9 | | Yes |  | Multiple sclerosis |  |  |  |  |  |
| G102 | 39 | | Yes |  | Brain aneurysms |  |  |  |  |  |
| G103 | 77 | | Yes |  | Cancer |  |  |  |  |  |
| G104 | 3 | | Yes |  | Kidney and heart disease |  |  |  |  |  |
| G108 | 18 | | Yes |  | Epilepsy |  |  |  |  |  |
| G109 | 20 | | Unclear | Unclear |  | Spine surgery |  |  |  |  |
| G110 | 58 | | Yes | Yes | Hydrocephalus |  | Pediatric |  |  | Family or caregivers |
| G111 | 65 | | Yes | Yes | Hydrocephalus |  | Pediatric |  |  |  |
| G112 | 63 | | Yes | Yes | Hydrocephalus |  | Adults |  |  |  |
| G113 | 67 | | Yes | Yes | Hydrocephalus |  | Adults |  |  | Family or caregivers |
| G114 | 16 | | Yes |  | Liver cancer |  |  |  |  |  |
| G115 | 13 | | Yes |  | Liver disease |  |  |  |  |  |
| G116 | 18 | | Yes |  | Cirrhosis |  |  |  |  |  |
| G118 | 19 | | Yes |  | Ulcerative colitis |  |  |  |  |  |
| G120 | 15 | | Yes |  | Prostate cancer |  |  | Males |  |  |
| G121 | 24 | | Yes |  | Breast cancer |  |  |  |  |  |
| G122 | 19 | | Yes |  |  |  |  |  |  |  |
| G123 | 27 | | Unclear | Unclear | Eye conditions |  | Pediatric |  |  | Family or caregivers |
| G127 | 24 | | Yes |  | Inflammatory bowel disease |  |  |  |  |  |
| G128 | 19 | | Unclear | Unclear |  |  |  |  |  |  |
| G131 | 14 | | Yes |  | Heart attack |  |  |  |  | Family or caregivers |
| G133 | 71 | | Yes | Yes | Diabetes |  |  |  |  |  |
| G135 | 4 | | Unclear | Unclear | Cancer |  |  |  |  |  |
| G138 | 113 | | Yes |  | Blood clots and pulmonary embolism |  |  |  |  |  |
| G140 | 18 | | Yes |  |  |  |  |  |  |  |
| G141 | 7 | | Yes |  |  |  |  |  |  |  |
| G148 | 10 | | Yes |  |  |  |  |  |  |  |
| G149 | 12 | | Unclear | Unclear |  |  |  |  |  | Medicare patients |
| G150 | 10 | | Yes |  |  |  |  |  |  |  |
| G153 | 11 | | Yes |  |  |  |  |  |  |  |
| G154 | 10 | | Unclear | Unclear |  |  |  |  |  |  |
| G160 | 22 | | Yes |  |  |  |  |  | Black, Indigenous and/or persons of color |  |
| G162 | 6 | | Yes |  |  |  |  |  |  |  |
| G164 | 24 | | Yes |  | Cancer |  |  |  |  |  |
| G165 | 8 | | Yes |  | Cancer |  |  |  |  |  |
| G166 | 10 | |  | Yes | Cancer |  |  |  |  |  |
| G167 | 24 | |  | Yes | Cancer |  |  |  |  |  |
| G168 | 4 | | Yes |  |  |  |  |  |  |  |
| G169 | 6 | | Yes |  |  |  |  |  |  |  |
| G170 | 33 | | Yes |  |  |  |  |  |  |  |
| G171 | 32 | | Yes |  |  |  |  |  |  |  |
| G172 | 27 | | Yes |  |  |  |  |  |  |  |
| G173 | 42 | | Yes |  |  |  |  |  |  | Family or caregivers |
| G175 | 13 | | Yes | Yes |  |  | Adults | Females |  |  |
| G176 | 12 | | Yes | Yes |  |  | Adults | Males |  |  |
| G179 | 28 | | Yes | Yes | Interstitial Lung Disease |  |  |  |  |  |
| G180 | 15 | | Yes |  | Heart conditions |  |  |  |  | Individuals with below average health literacy |
| G181 | 40 | | Yes |  | Coronary artery disease |  |  |  |  |  |
| G182 | 19 | | Yes |  | Heart disease and stroke |  |  |  |  |  |
| G183 | 21 | | Yes |  | Heart failure |  |  |  |  |  |
| G184 | 4 | |  | Yes | Diabetes |  |  |  |  |  |
| G185 | 9 | | Yes |  | Cancer |  |  |  |  |  |
| G186 | 32 | | Yes | Yes | Cancer |  |  |  |  |  |
| G188 | 5 | | Yes | Yes | Diabetes |  |  |  |  |  |
| G189 | 23 | | Yes | Yes | Behavioral health conditions |  |  |  |  |  |
| G190 | 5 | | Yes |  |  |  |  |  |  |  |
| G192 | 9 | | Yes |  |  |  |  |  |  |  |
| G193 | 7 | | Yes |  |  |  |  |  |  |  |
| G194 | 8 | | Yes |  |  | Bone marrow transplant surgery |  |  |  |  |
| G195 | 8 | | Yes |  |  |  | Pediatric |  |  | Family or caregivers |
| G196 | 10 | | Yes |  | Uterine fibroids |  |  | Females |  |  |
| G197 | 25 | | Yes |  |  |  |  |  |  |  |
| G198 | 64 | | Yes | Yes | Cancer |  |  |  |  |  |
| G199 | 97 | | Yes |  |  |  |  |  |  |  |
| G200 | 10 | | Yes |  |  |  |  |  |  |  |
| G202 | 15 | | Unclear | Unclear |  | Any operation |  |  |  |  |
| G203 | 11 | | Yes |  |  |  | Adults |  |  |  |
| G204 | 7 | | Yes |  |  |  |  |  |  |  |
| G205 | 25 | | Yes |  | Cancer |  |  |  |  |  |
| G207 | 37 | | Yes |  |  |  | Older adults |  |  |  |
| G208 | 7 | | Yes |  |  |  |  |  |  |  |
| G209 | 11 | |  | Yes | Cancer |  |  |  |  | Family or caregivers |
| G210 | 15 | | Yes |  | Kidney disease |  |  |  |  |  |
| G211 | 15 | | Yes |  | Heart disease |  |  | Females | Black or African American |  |
| G212 | 15 | |  | Yes | Substance use disorder and addiction |  |  |  |  |  |
| G213 | 5 | | Yes |  | Primary immune deﬁciency diseases |  |  |  |  |  |
| G214 | 13 | | Yes |  |  |  | Older adults |  |  |  |
| G215 | 13 | |  | Yes | Cancer |  |  |  |  |  |
| G216 | 15 | | Yes |  | Cancer |  |  |  |  |  |
| G217 | 15 | | Yes | Yes | Chronic obstructive pulmonary disease |  |  |  |  | Family or caregivers |
| G218 | 19 | | Yes |  | Eye conditions |  |  |  |  |  |
| G221 | 8 | | Yes |  | Breast cancer |  |  |  |  |  |
| G224 | 12 | | Yes |  |  |  |  |  |  |  |
| G225 | 55 | | Yes |  |  |  |  |  |  |  |
| G227 | 4 | | Yes |  |  |  |  |  |  |  |
| G228 | 25 | | Yes |  |  |  |  |  |  |  |
| G229 | 50 | | Yes | Yes | Cancer |  |  |  |  |  |
| G230 | 17 | | Yes |  | Concussion |  | Pediatric |  |  | Family or caregivers |
| G231 | 31 | | Yes |  | Heart disease |  |  | Females |  |  |
| G232 | 72 | | Yes |  | Heart disease |  |  | Females |  |  |
| G233 | 11 | | Yes |  | Chronic obstructive pulmonary disease |  |  |  |  |  |
| G234 | 8 | | Yes |  | Celiac disease |  |  |  |  |  |
| G235 | 27 | |  | Yes | Multiple Sclerosis |  |  |  |  |  |
| G236 | 6 | | Yes |  | Inflammatory bowel disease |  |  |  |  |  |
| G237 | 13 | | Yes |  | Dementia |  | Older adults |  |  |  |
| G239 | 10 | | Yes |  | Idiopathic hypersomnia |  |  |  |  |  |
| G241 | 10 | | Yes |  | Non-small cell lung cancer |  |  |  |  |  |
| G242 | 10 | | Yes |  | Heart failure |  |  |  |  |  |
| G243 | 15 | | Yes |  | Hyperthyroidism |  |  |  |  |  |
| G244 | 18 | | Yes |  | Schizophrenia |  |  |  |  | Family or caregivers |
| G245 | 9 | | Yes |  | Ulcerative colitis |  |  |  |  |  |
| G246 | 10 | | Yes |  | Osteoarthritis |  |  |  |  |  |
| G247 | 10 | | Yes |  | Kidney failure |  |  |  |  |  |
| G248 | 10 | | Yes |  | Asthma |  |  |  |  |  |
| G249 | 14 | | Yes |  | Skin allergy |  |  |  |  |  |
| G250 | 13 | | Yes |  | Hyperhidrosis |  |  |  |  |  |
| G251 | 10 | | Yes |  | Psoriasis |  |  |  |  |  |
| G252 | 17 | | Yes |  | Melanoma |  |  |  |  |  |
| G253 | 17 | | Yes |  | HIV/AIDS |  |  |  |  |  |
| G254 | 19 | |  | Yes | Ovarian cancer |  |  |  |  |  |
| G255 | 13 | | Yes |  | Stroke |  |  |  |  |  |
| G256 | 11 | | Yes |  | Pulmonary arterial hypertension |  |  |  |  |  |
| G257 | 9 | | Yes |  | Depression |  |  |  |  |  |
| G258 | 13 | | Yes |  | Asthma |  | Pediatric |  |  | Family or caregivers |
| G259 | 13 | | Yes |  | Parkinson’s |  |  |  |  |  |
| G260 | 12 | | Yes |  | Alzheimer's |  | Older adults |  |  |  |
| G261 | 15 | |  | Yes | Breast cancer |  |  |  |  |  |
| G262 | 8 | | Yes |  | Polycythemia vera |  |  |  |  |  |
| G264 | 12 | | Yes |  | Migraines |  |  |  |  |  |
| G265 | 13 | | Yes |  | Pelvic Inflammatory Disease |  |  | Females |  |  |
| G266 | 13 | | Yes |  | Cancer |  |  |  |  |  |
| G267 | 10 | |  | Yes | Treatment Resistant Depression |  |  |  |  |  |
| G268 | 12 | | Yes |  | Urinary incontinence |  |  | Males |  |  |
| G269 | 11 | | Yes |  | Pelvic pain |  |  |  |  |  |
| G270 | 11 | |  | Yes | Menopause |  |  | Females |  |  |
| G271 | 5 | | Yes |  |  |  |  |  |  |  |
| G272 | 11 | | Yes |  | Fibromyalgia and Myalgic Encephalomyelitis |  |  |  |  |  |
| G273 | 11 | | Yes |  | Bipolar disorder |  |  |  |  |  |
| G274 | 14 | | Yes |  | Endometriosis |  |  | Females |  |  |
| G275 | 11 | | Yes | Yes | Atrial fibrillation |  |  |  |  |  |
| G276 | 29 | | Yes | Yes | Genital herpes |  |  |  |  |  |
| G277 | 18 | | Yes |  | HER2- positive breast cancer |  |  |  |  |  |
| G278 | 11 | | Yes |  | Gastroesophageal reflux disease |  |  |  |  |  |
| G279 | 11 | | Yes | Yes | Chronic heart failure |  |  |  |  |  |
| G280 | 12 | | Yes |  | Pulmonary arterial hypertension |  |  |  |  |  |
| G281 | 12 | | Yes |  | Multiple Sclerosis |  |  |  |  |  |
| G282 | 4 | | Yes |  | Cancer |  | Pediatric |  |  | Family or caregivers |
| G283 | 27 | | Yes | Yes | Attention-deficit/hyperactivity disorder |  |  |  |  |  |
| G284 | 21 | | Yes | Yes | Anorexia Nervosa |  |  |  |  |  |
| G285 | 18 | | Yes | Yes | Binge Eating Disorder |  |  |  |  |  |
| G286 | 27 | | Yes | Yes | Bipolar disorder |  |  |  |  |  |
| G287 | 27 | | Yes | Yes | Borderline Personality Disorder |  |  |  |  |  |
| G288 | 20 | | Yes | Yes | Bulimia Nervosa |  |  |  |  |  |
| G289 | 25 | | Yes | Yes | Depression |  |  |  |  |  |
| G290 | 13 | | Yes | Yes | Generalized Anxiety Disorder |  |  |  |  |  |
| G291 | 30 | | Yes | Yes | Obsessive-compulsive disorder |  |  |  |  |  |
| G292 | 23 | | Yes | Yes | Opioid Addiction |  |  |  |  |  |
| G293 | 20 | | Yes | Yes | Orthorexia |  |  |  |  |  |
| G294 | 16 | | Yes | Yes | Panic Disorder |  |  |  |  |  |
| G295 | 29 | | Yes | Yes | Post-traumatic stress disorder |  |  |  |  |  |
| G296 | 27 | | Yes | Yes | Schizophrenia |  |  |  |  |  |
| G297 | 18 | | Yes | Yes | Social Anxiety Disorder |  |  |  |  |  |
